# Prediction of cccDNA dynamics in hepatitis B patients by a combination of serum surrogate markers

**DOI:** 10.1371/journal.pcbi.1012615

**Published:** 2025-01-09

**Authors:** Kwang Su Kim, Masashi Iwamoto, Kosaku Kitagawa, Hyeongki Park, Sanae Hayashi, Senko Tsukuda, Takeshi Matsui, Masanori Atsukawa, Kentaro Matsuura, Natthaya Chuaypen, Pisit Tangkijvanich, Lena Allweiss, Takara Nishiyama, Naotoshi Nakamura, Yasuhisa Fujita, Eiryo Kawakami, Shinji Nakaoka, Masamichi Muramatsu, Kazuyuki Aihara, Takaji Wakita, Alan S. Perelson, Maura Dandri, Koichi Watashi, Shingo Iwami, Yasuhito Tanaka

**Affiliations:** 1 interdisciplinary Biology Laboratory (iBLab), Division of Natural Science, Graduate School of Science, Nagoya University, Nagoya, Japan; 2 Department of Scientific Computing, Pukyong National University, Busan, South Korea; 3 Department of Virology II, National Institute of Infectious Diseases, Tokyo, Japan; 4 Department of Gastroenterology and Hepatology, Faculty of Life Sciences, Kumamoto University, Kumamoto, Japan; 5 Nuffield Department of Medicine, University of Oxford, Oxford, United Kingdom; 6 Center for Gastroenterology, Teine Keijinkai Hospital, Sapporo, Japan; 7 Department of Gastroenterology and Hepatology, Nippon Medical School, Tokyo, Japan; 8 Department of Gastroenterology and Metabolism, Nagoya City University, Graduate School of Medical Sciences, Nagoya, Japan; 9 Center of Excellence in Hepatitis and Liver Cancer, Department of Biochemistry, Faculty of Medicine, Chulalongkorn University, Bangkok, Thailand; 10 Department of Internal Medicine, University Medical Center Hamburg-Eppendorf, Hamburg, Germany; 11 German Center for Infection Research (DZIF), Hamburg-Lübeck-Borstel-Riems partner sites, Germany; 12 Artificial Intelligence Medicine, Graduate School of Medicine, Chiba University, Chiba, Japan; 13 Medical Sciences Innovation Hub Program; RIKEN, Yokohama, Kanagawa, Japan; 14 Faculty of Advanced Life Science, Hokkaido University, Sapporo, Japan; 15 International Research Center for Neurointelligence, The University of Tokyo Institutes for Advanced Study, The University of Tokyo, Tokyo, Japan; 16 Theoretical Biology and Biophysics Group, Los Alamos National Laboratory, Los Alamos, New Mexico, United States of America; 17 Research Center for Drug and Vaccine Development, National Institute of Infectious Diseases, Tokyo, Japan; 18 Department of Applied Biological Sciences, Faculty of Science and Technology, Tokyo University of Sciences, Chiba, Japan; 19 Institute of Mathematics for Industry, Kyushu University,; Fukuoka, Japan; 20 Institute for the Advanced Study of Human Biology (ASHBi), Kyoto University, Kyoto, Japan; 21 NEXT-Ganken Program, Japanese Foundation for Cancer Research (JFCR), Tokyo, Japan; 22 Interdisciplinary Theoretical and Mathematical Sciences (iTHEMS), RIKEN, Wako, Japan; 23 Science Groove Inc., Fukuoka, Japan; Pennsylvania State University, UNITED STATES OF AMERICA

## Abstract

Quantification of intrahepatic covalently closed circular DNA (cccDNA) is a key for evaluating an elimination of hepatitis B virus (HBV) in infected patients. However, quantifying cccDNA requires invasive methods such as a liver biopsy, which makes it impractical to access the dynamics of cccDNA in patients. Although HBV RNA and HBV core-related antigens (HBcrAg) have been proposed as surrogate markers for evaluating cccDNA activity, they do not necessarily estimate the amount of cccDNA. Here, we employed a recently developed multiscale mathematical model describing intra- and intercellular viral propagation and applied it in HBV-infected patients under treatment. We developed a model that can predict intracellular HBV dynamics by use of extracellular viral markers, including HBsAg, HBV DNA, and HBcrAg in peripheral blood. Importantly, the model prediction of the amount of cccDNA in patients over time was confirmed to be well correlated with the data for quantified cccDNA by paired liver biopsy. Thus, our method combining classic and emerging surrogate markers enables us to predict the decay dynamics of cccDNA in patients undergoing treatment.

## Author summary

Chronic hepatitis B (CHB) poses a major global health issue due to the persistence of covalently closed circular DNA (cccDNA) in the liver, which acts as a template for ongoing viral replication. Current therapies, such as PEG IFN-α, could reduce cccDNA levels but rarely achieve complete elimination. In this study, we developed and applied a multiscale mathematical model that combines data from in vitro, in vivo, and clinical studies to predict the dynamics of cccDNA in CHB patients during treatment. Our model uses extracellular viral markers—HBV DNA, HBsAg, and HBcrAg—to estimate cccDNA levels, potentially reducing the reliance on invasive liver biopsies. We demonstrated that our model may predict cccDNA turnover under treatment and can suggest options such as prolonging the duration of treatment to patients who get a large reduction in cccDNA to achieve a complete cure. This approach provides a new tool for personalizing CHB treatment strategies, aiming to achieve a complete cure by targeting and monitoring cccDNA dynamics more effectively.

## Introduction

Chronic infection with hepatitis B virus (HBV) elevates the risk of developing hepatocellular carcinoma. The WHO has estimated that 297 million people worldwide are living with HBV and that 820,000 people died from this infection in 2019 (https://www.who.int/en/news-room/fact-sheets/detail/hepatitis-b) [1,2]. Persistence of HBV infection is attributable to the formation of covalently closed circular DNA (cccDNA) in the nucleus of an infected hepatocyte. The cccDNA acts as a reservoir that transcribes HBV RNA and produces HBV DNA through reverse transcription. The cccDNA also drives transcription for viral proteins such as HBV surface antigen (HBsAg) and HBV core-related antigen (HBcrAg). HBsAg makes up the HBV envelope and is secreted into the serum as a virion particle or alone as a subviral particle. HBcrAg comprises HBV core antigen (HBcAg) that forms a viral capsid that serves as a scaffold for reverse transcription to HBV DNA, and also nonstructural proteins such as, HBV e antigen (HBeAg), and a 22-kDa truncated core-related protein (p22cr) [1,3]. HBV DNA integrated in cellular chromosomes is an additional source of HBV antigens, especially HBsAg [4,5].

Pegylated interferon alpha (PEG IFN-α) and nucleos(t)ide analogues (NAs) are used to treat chronic hepatitis B (CHB) [6]. PEG IFN-α activates host immune responses and suppresses viral replication. NAs inhibit reverse transcription, thus reducing the formation of HBV DNA and improving liver pathology [7]. In most patients, these therapies typically lower serum HBV DNA to undetectable levels, but they have limited impact on HBV antigens like HBsAg. Patients often remain positive for HBsAg, which is considered *a partial cure*. These treatments have limited potential to achieve *a functional cure*, defined as undetectable HBV DNA and HBsAg in the serum, along with cccDNA silencing, with or without HBeAg seroconversion [8,9]. Note that HBeAg seroconversion signifies the emergence of HBeAg negativity and anti-HBeAg antibody, meaning that immune control of HBV infection is achieved. This is observed in some CHB patients during or after anti-HBV treatment and marks stable remission of HBV infection [10]. Functional cure is a current clinical goal of anti-HBV therapy. A *complete cure*, that is, undetectable HBV DNA and HBsAg in the serum and cccDNA clearance in the liver is the eventual goal for HBV elimination. Because of the difficulty of transcriptional silencing and elimination of cccDNA, patients often require lifelong treatment and few maintain sustained viral or clinical remission off therapy [11].

To evaluate the elimination of cccDNA, the amount of cccDNA in a patient’s liver needs to be quantified by liver biopsy, which is not generally done in clinical practice. Surrogate markers are used instead. Ideal surrogate markers for evaluating CHB treatment should be specific, sensitive, and noninvasive and should reflect amounts or dynamics of cccDNA in the liver [12]. The level of HBsAg in the serum under treatment is reported to have a correlation with cccDNA and HBV DNA in the cells, which means it is known as a classic surrogate marker for cccDNA [13]. However, it has been shown to have only a weak or no correlation with cccDNA, especially in HBeAg-negative patients [4,14]. Because HBsAg is not produced from persistent cccDNA transcription but from integrated HBV DNA genomes, increasing research suggests that the levels of HBV RNA and HBcrAg in the serum may offer better reflections of transcriptionally active cccDNA in the liver as emerging surrogate markers [15–18]. This is because they are not generated by integrated viral DNA. However, the expression of HBV RNA and HBcrAg relies not only on the quantity of cccDNA but also on its transcriptional activity, which can vary within a patient cohort due to factors such as disease phase and whether patients are undergoing antiviral therapy, leading to significant interindividual variation [19,20]. Therefore, these viral markers may not be particularly useful for predicting cccDNA decay. Essentially, it is challenging to determine whether the reduction in these surrogate markers during treatment is a result of cccDNA inactivation or decay. The absence of a method for monitoring intrahepatic cccDNA dynamics, rather than its activity, obstructs the assessment of complete cure status and adds to the difficulty in forecasting viral relapse post-treatment.

In this study, we employed a multiscale mathematical model that recently developed by our group based on both *in vitro* experiments using primary human hepatocytes (PHH) and *in vivo* experiments using humanized mice for HBV infection [21]. Our method uses two classic surrogate markers, HBsAg and HBV DNA, in addition to one emerging surrogate marker, HBcrAg, which can be easily detected and are highly specific among the emerging surrogate markers [1] for estimating the intrahepatic cccDNA dynamics. We demonstrated that our model derived from the multiscale mathematical model can be applied to clinical data during treatment in both HBeAg-positive and -negative patient cohorts, and that it can estimate the turnover of cccDNA and predict the cccDNA level as confirmed by paired liver biopsy. In other words, in this method, if the baseline cccDNA amount prior to treatment has been quantified by liver biopsy, it is possible to estimate not only the decay of cccDNA but also the amount of cccDNA during treatment, which eliminates the need to repeat liver biopsies for achieving complete cure.

## Results

**Fig 1:**
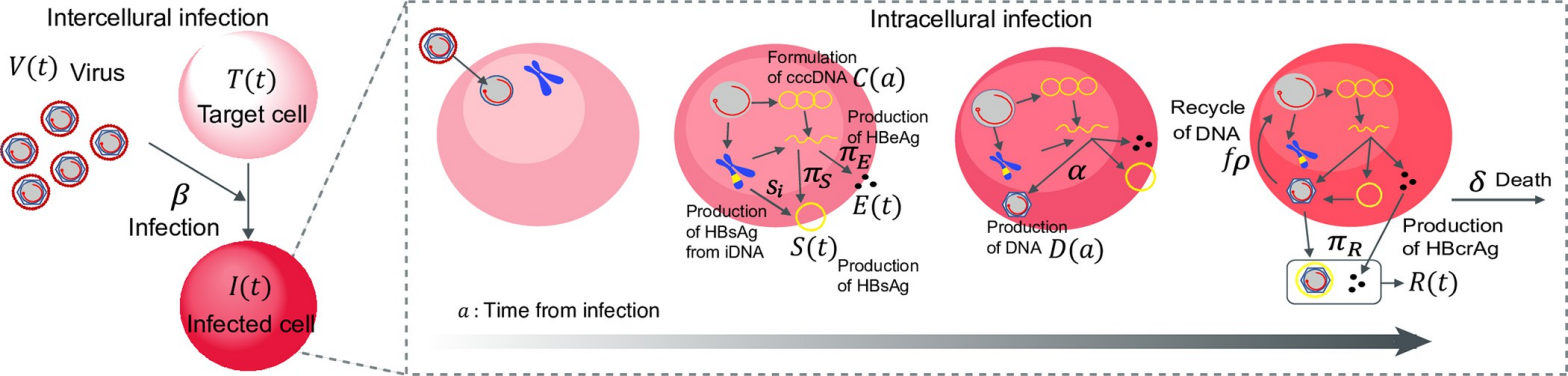
Schematic diagram of intra-, inter- and extra-cellular virus dynamics. HBV virion that enters its target cell forms cccDNA in the nucleus and generates intracellular HBV DNA at a rate *α*. The antigens HBsAg, HBeAg, and HBcrAg are produced from cccDNA at rates *π*_*S*_,*π*_*E*_, and *π*_*R*_, respectively, and are cleared from the peripheral blood at a rate *σ*. Additionally, HBsAg may also be produced by integrated HBV DNA (iDNA) within infected cells at a rate *s*_*i*_. Intracellular HBV DNA is consumed at a rate *ρ*, with a fraction 1−*f* of this DNA assembling with viral proteins to form virus particles, which are then exported from the infected cells, while the remaining fraction *f* is reused to form more cccDNA, which has a degradation rate of *d*. The infected cells die at rate *δ*, and the exported viral particles, which are cleared at a rate *μ*, infect target cells at rate *β*.

We first here introduce a multiscale model using partial differential equations (PDEs) that couple intra-, inter- and extra-cellular virus dynamics for analyzing multiscale experimental data of HBV infection (c.f. [22])(**Fig 1**):

$$\frac{dT(t)}{dt}=s-d_{T}T(t)-\beta T(t)V(t),$$ (1)

$$(\frac{\partial }{\partial t}+\frac{\partial }{\partial a})i(t,a)=-\delta i(t,a),$$ (2)

$$\frac{dV(t)}{dt}=(1-f)\rho \int_{0}^{\infty }D(a)i(t,a)da-\mu V(t),$$ (3)

$$\frac{dS(t)}{dt}=\pi_{S}\int_{0}^{\infty }C(a)i(t,a)da+s_{i}\int_{0}^{\infty }i(t,a)da-\sigma S(t),$$ (4)

$$\frac{dR(t)}{dt}=\pi_{R}\int_{0}^{\infty }C(a)i(t,a)da-\sigma R(t),$$ (5)

$$\frac{dC(a)}{da}=f\rho D(a)-dC(a),$$ (6)

$$\frac{dD(a)}{da}=\alpha C(a)-\rho D(a).$$ (7)

with the boundary condition *i*(*t*,0) = *βT*(*t*)*V*(*t*) and initial condition *i*(0,*a*) = *i*_0_(*a*). The intercellular variables *T*(*t*) and *V*(*t*) are the number of uninfected cells and the extracellular HBV DNA, respectively. We defined the density of infected cells with infection age *a* as *i*(*t*,*a*), and therefore the total number of infected cells is $I(t)=\int_{0}^{\infty }i(t,a)da$. The intracellular variables *C*(*a*) and *D*(*a*), which evolve depending on the age *a*, represent the amount of intracellular cccDNA and HBV DNA, respectively. We also defined extracellular variables used as “extracellular viral markers” to predict the dynamics of cccDNA in hepatocytes, that is, the amount of HBsAg and HBcrAg antigens as *S*(*t*) and *R*(*t*), respectively. The definition of an age-structured population model is found in [23].

We here assumed target cells, *T*, are supplied at rate *s*, die at per capita rate *d*_*T*_, are infected by viruses at rate *β*, and the infected cells die at per capita rate *δ*. We also assumed that HBsAg and HBcrAg antigens are produced from cccDNA in infected cells at rates *π*_*S*_ and *π*_*R*_, and are cleared at rate *σ*, respectively. Additionally, HBsAg may also be produced by integrated HBV DNA (iDNA) in the infected cells at a rate *s*_*i*_. The exported viral particles, i.e., extracellular HBV DNA load, is assumed to be cleared at rate *μ* per virion. The entry virion forms cccDNA in the nucleus and produces intracellular HBV DNA at rate *α*. The intracellular HBV DNA is consumed at rate *ρ*, of which a fraction 1−*f* of HBV DNA assembled with viral proteins as virus particles are exported from infected cells and the other fractin *f* is reused for further cccDNA formation having a degradation rate of *d*.

<

### Linearizing multiscale model under treatments

As in our previously reported [21], the mathematical model (i.e., Eqs 1–7) can be transformed into linear ODE model under suitable assumptions (see **Note A** in **S1 Text**).

### (i) Linearized equations under potent NAs treatment

We assumed that NAs treatment is potent enough that intracellular HBV replications and *de novo* infections are negligible after treatment initiation [24–27], i.e., the antiviral effectiveness of NAs on intracellular HBV replications is assumed to be 0<*ε*≤1 and

$$i(t,a)=\left\{ \begin{array}{cc} 0 & t>a\\ i_{0}(a) & t<a \end{array}\right..$$

Then S2-S8 Eqs (**Note A** in **S1 Text**) can be simplified as follows:

$$\frac{dI(t)}{dt}=-\delta I(t),$$ (8)

$$\frac{dV(t)}{dt}=(1-f)\rho DD(t)-\mu V(t),$$ (9)

$$\frac{dS(t)}{dt}=\pi_{S}CC(t)+s_{i}I(t)-\sigma S(t),$$ (10)

$$\frac{dR(t)}{dt}={(1-m)\pi }_{R}CC(t)-\sigma R(t),$$ (11)

$$\frac{dCC(t)}{dt}=f\rho DD(t)-(d+\delta )CC(t),$$ (12)

$$\frac{dDD(t)}{dt}=(1-\varepsilon )\alpha CC(t)-(\rho +\delta )CC(t).$$ (13)

Here we assume that all variables in Eqs 8–13 are in steady state before treatment initiation [28], and particularly that the infected cells obtain a stable age distribution, i.e., *i*_0_(*a*) = *βT*(0)*V*(0)*e*^−*δa*^. Note that parameter *m* indicates the effect of "precore mutation" and "basic core promoter mutations", which sometimes occurs in clinical settings after treatments that reduce the production of HBeAg [29–31]. Because the viral marker of HBcrAg includes HBeAg, these mutations affect and alter the dynamics of HBcrAg.

Since Eqs 8–13 are a set of linear ODEs, we directly solve them, and find the following analytical solutions:

$$V(t)=V(0)(Ae^{(\lambda_{1}-\delta )t}+Be^{(\lambda_{2}-\delta )t}+(1-A-B)e^{-\mu t}),$$ (14)

$$S(t)=S(0)(Ce^{(\lambda_{1}-\delta )t}+De^{(\lambda_{2}-\delta )t}+Ee^{-\delta t}+(1-C-D-E)e^{-\sigma t}),$$ (15)

$$R(t)=R(0)(C^{\prime }e^{(\lambda_{1}-\delta )t}+D^{\prime }e^{(\lambda_{2}-\delta )t}+(1-C^{\prime }-D^{\prime })e^{-\sigma t}).$$ (16)

Here $A=\frac{-\{{(\lambda_{1}+d+\delta )\lambda }_{2}+\delta \rho \}\mu }{\left(\lambda_{1}-\delta +\mu )(\lambda_{1}-\lambda_{2})(d+\delta \right)}$, $B=\frac{\{{(\lambda_{2}+d+\delta )\lambda }_{1}+\delta \rho \}\mu }{\left(\lambda_{2}-\delta +\mu )(\lambda_{1}-\lambda_{2})(d+\delta \right)},$ $C=\frac{-(\lambda_{2}-\delta )(1-x)\sigma }{\left(\lambda_{1}-\delta +\sigma )(\lambda_{1}-\lambda_{2}\right)},$ $D=\frac{(\lambda_{1}-\delta )(1-x)\sigma }{\left(\lambda_{2}-\delta +\sigma )(\lambda_{1}-\lambda_{2}\right)},$ $E=\frac{\sigma x}{\sigma -\delta },\;C^{\prime }=\frac{-(1-m)(\lambda_{2}-\delta )\sigma }{\left(\lambda_{1}-\delta +\sigma )(\lambda_{1}-\lambda_{2}\right)},$ $D^{\prime }=\frac{(1-m)(\lambda_{1}-\delta )\sigma }{\left(\lambda_{2}-\delta +\sigma )(\lambda_{1}-\lambda_{2}\right)}$ and $\lambda_{1,2}=\frac{-(\rho +d)\pm \sqrt{{\left(\rho -d\right)}^{2}+4f(1-\varepsilon )\alpha \rho }}{2}$. Note that *x* is the proportion of HBsAg produced from integrated DNA: $x=\frac{s_{i}I(0)}{\pi_{s}CC(0)+s_{i}I(0)}$.

### (ii) Linearized equations under potent PEG IFN-α treatment

We also assumed that PEG IFN-α treatment is potent enough that intracellular HBV replication and *de novo* infections are negligible after treatment initiation [25,26,32–34], i.e., the antiviral effect of PEG IFN-α on intracellular HBV replications is assumed to be 0<*ε*≤1 and

$$i(t,a)=\left\{ \begin{array}{cc} 0 & t>a\\ i_{0}(a) & t<a \end{array}.\right.$$

**Fig 2:**
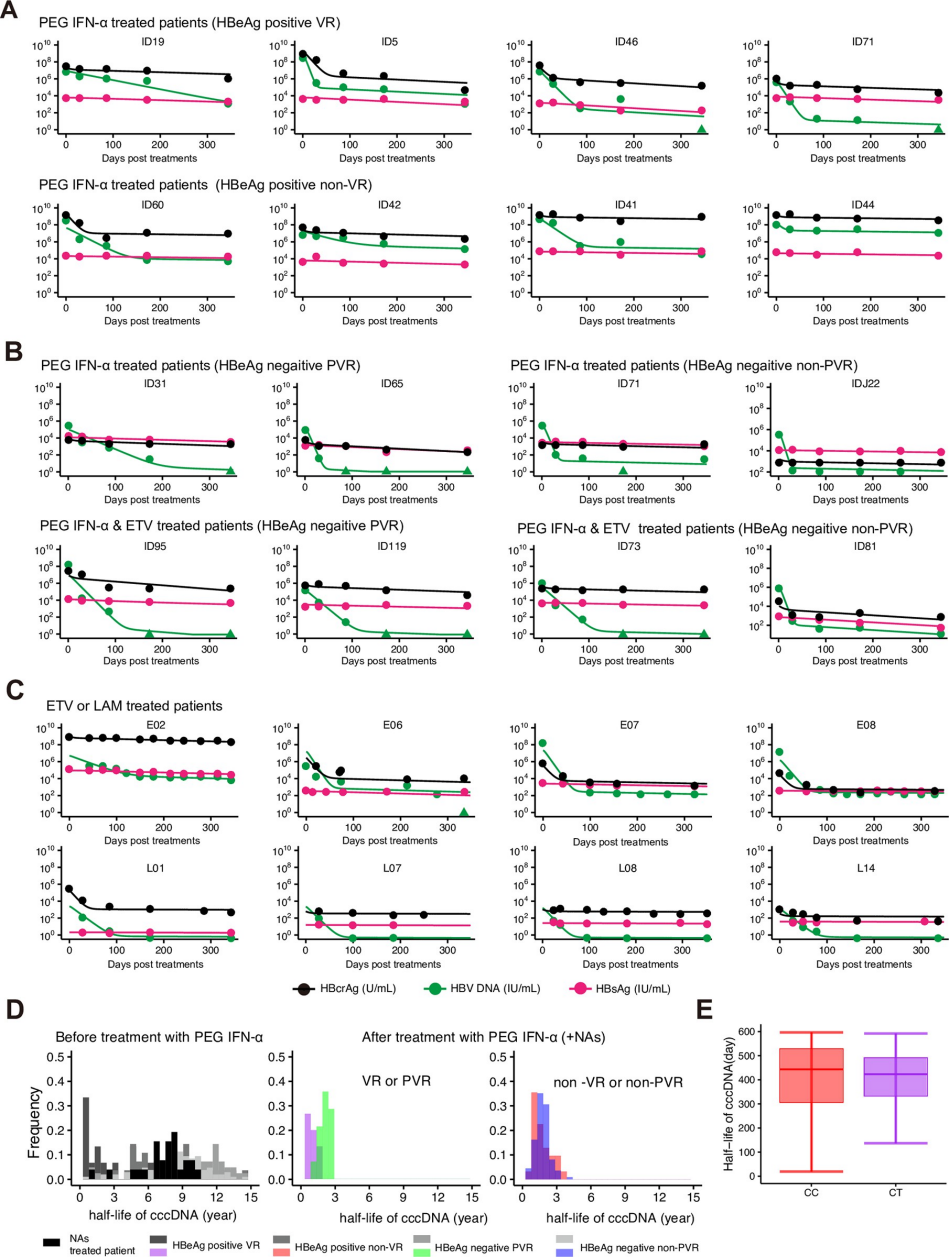
**Dynamics of HBV infection in patients treated with PEG IFN-α:** Representative decay characteristics are shown for extracellular HBV DNA, HBsAg, and HBcrAg in peripheral blood of **(A)** HBeAg-positive patients treated with PEG IFN-α with VR or non-VR, **(B)** HBeAg-negative patients treated with PEG IFN-α with PVR or non-PVR, and HBeAg-negative patients treated with PEG IFN-α and ETV with PVR or non-PVR, and **(C)** patients treated with ETV or LAM. **(D)** The distributions of the half-life of cccDNA before and after treatment with PEG IFN-α (+ETV or LAM) for HBeAg-positive/negative and (P)VR/non-(P)VR patients are shown. **(E)** Estimated half-life of cccDNA in hepatocyte from patients with IL28B CC (n = 208) or CT (n = 18) genotype treated with PEG IFN-α are shown. Black line indicates the median; box and whiskers show the interquartile range (IQR) and 1.5xIQR, respectively.

It has been reported that PEG IFN-α induces interferon-stimulated genes (ISGs) and ISGs potentially degrade intracellular cccDNA. Therefore, we assumed PEG IFN-α increases the cccDNA degradation rate [35], i.e., *d*_*IFN*_(>*d*). In addition, because PEG IFN-α may enhance the decay rate of infected cells in HBV infection *in vivo* due to cytotoxic effects (but relatively mild), we assumed *δ*_*IFN*_(>*δ*) in the data fitting (**Fig 2A, 2B, 2C,** and **B** in **S1 Text**). Then S2-S8 Eqs (**Note A** in **S1 Text**) can be simplified to

$$\frac{dI(t)}{dt}=-\delta_{IFN}I(t),$$ (17)

$$\frac{dV(t)}{dt}=(1-f)\rho DD(t)-\mu V(t),$$ (18)

$$\frac{dS(t)}{dt}=\pi_{S}CC(t)+s_{i}I(t)-\sigma S(t),$$ (19)

$$\frac{dR(t)}{dt}={(1-m)\pi }_{R}CC(t)-\sigma R(t),$$ (20)

$$\frac{dCC(t)}{dt}=f\rho DD(t)-(d_{IFN}+\delta_{IFN})CC(t),$$ (21)

$$\frac{dDD(t)}{dt}=(1-\varepsilon )\alpha CC(t)-(\rho +\delta_{IFN})CC(t).$$ (22)

We also assumed the inhibition rate of HBcrAg production, *m*, and all variables in Eqs 17–22 are in steady state before treatment initiation, and that the infected cells have obtained a stable age distribution, i.e., *i*_0_(*a*) = *βT*(0)*V*(0)*e*^−*δa*^. Therefore, by solving Eqs 17–22, we find

$$V(t)=V(0)(A_{IFN}e^{(\eta_{1}-\delta_{IFN})t}+B_{IFN}e^{(\eta_{2}-\delta_{IFN})t}+(1-A_{IFN}-B_{IFN})e^{-\mu t}),$$ (23)

$$S(t)=S(0)(C_{IFN}e^{(\eta_{1}-\delta_{IFN})t}+D_{IFN}e^{(\eta_{2}-\delta_{IFN})t}+E_{IFN}e^{-\delta_{IFN}t}+(1-C_{IFN}-D_{IFN}-E_{IFN})e^{-\sigma t}),$$ (24)

$$R(t)=R(0)(C_{IFN}^{\prime }e^{(\eta_{1}-\delta_{IFN})t}+D_{IFN}^{\prime }e^{(\eta_{2}-\delta_{IFN})t}+(1-C_{IFN}^{\prime }-D_{IFN}^{\prime })e^{-\sigma t}).$$ (25)

Similarly, the total amount of cccDNA *CC*(*t*) and the amount of cccDNA per infected cell $\tilde{C}(t)=CC(t)/I(t)$ are derived as follows:

$$CC(t)=CC(0)(Z_{IFN}e^{(\eta_{1}-\delta_{IFN})t}+(1-Z_{IFN})e^{(\eta_{2}-\delta_{IFN})t}),$$ (26)

$$\tilde{C}(t)=\tilde{C}(0)(Z_{IFN}e^{\eta_{1}t}+(1-Z_{IFN})e^{\eta_{2}t}),$$ (27)

where $A_{IFN}=\frac{-\{{(\eta_{1}+d+\delta )\eta }_{2}+(d-d_{IFN}+\delta )\rho \}\mu }{\left(\eta_{1}-\delta_{IFN}+\mu )(\eta_{1}-\eta_{2})(d+\delta \right)},$ $B_{IFN}=\frac{\{{(\eta_{2}+d+\delta )\eta }_{1}+(d-d_{IFN}+\delta )\rho \}\mu }{\left(\eta_{2}-\delta_{IFN}+\mu )(\eta_{1}-\eta_{2})(d+\delta \right)},$ $C_{IFN}=\frac{-(\eta_{2}-d+d_{IFN}-\delta )(1-x)\sigma }{\left(\eta_{1}-\delta_{IFN}+\sigma )(\eta_{1}-\eta_{2}\right)},$ $D_{IFN}=\frac{(\eta_{1}-d+d_{IFN}-\delta )(1-x)\sigma }{\left(\eta_{2}-\delta_{IFN}+\sigma )(\eta_{1}-\eta_{2}\right)},$ $E_{IFN}=\frac{\sigma x}{\sigma -\delta_{IFN}},$ $C_{IFN}^{\prime }=\frac{-(1-m)(\eta_{2}-d+d_{IFN}-\delta )\sigma }{\left(\eta_{1}-\delta_{IFN}+\sigma )(\eta_{1}-\eta_{2}\right)},$ $D_{IFN}^{\prime }=\frac{(1-m)(\eta_{1}-d+d_{IFN}-\delta )\sigma }{\left(\eta_{2}-\delta_{IFN}+\sigma )(\eta_{1}-\eta_{2}\right)},$ $Z_{IFN}=\frac{{-\eta }_{2}+d-d_{IFN}+\delta }{\eta_{1}-\eta_{2}}$ and $\eta_{1,2}=\frac{-(d_{IFN}+\rho )\pm \sqrt{{\left(d_{IFN}-\rho \right)}^{2}+4f(1-\varepsilon )\alpha \rho }}{2}$. Note that *x* is the proportion of HBsAg produced from integrated DNA: $x=\frac{s_{i}I(0)}{\pi_{S}CC(0)+s_{i}I(0)}$.

### Quantifying intrahepatic cccDNA dynamics in chronically HBV-infected patients

We analyzed CHB cohorts comprising a total of 226 patients in three Japanese and one Thailand hospitals, among whom 199 patients were treated with PEG IFN-α monotherapy or PEG IFN-α combination therapy with NAs (ETV or lamivudine [LAM]) for 48 weeks and 27 patients received NAs. Serum samples from these patients were collected from the start of treatment (day 0) to the end of treatment (48 weeks) to detect HBcrAg, HBV DNA, and HBsAg (**Fig A** in **S1 Text**). We separated the patients into four groups according to their HBeAg status and their eventual virological response to treatment. *Virological response* (VR) was defined as HBeAg clearance and HBV DNA level <2,000 IU/ml at 48 weeks after treatment in HBeAg-positive CHB. *Persistent VR* (PVR) was defined as HBeAg clearance and HBV DNA level <2,000 IU/ml at 96 weeks after treatment in HBeAg-negative CHB. Non-VR and non-PVR were those who did not reach the criteria for VR and PVR, respectively. We analyzed the following longitudinally monitored viral markers in peripheral blood [36,37]: extracellular HBV DNA and HBsAg as classic surrogate markers as well HBcrAg as an emerging surrogate marker that can be detected specifically and easily by enzyme immunoassay, for up to 48 weeks after starting treatment (**Fig A** in **S1 Text** and **Methods**). Patients treated with PEG IFN-α showed either a single-phase or biphasic reduction of HBV DNA (**Fig 2A** and **2B**). In contrast, the decrease in HBsAg and HBcrAg varied among patients, with some showing a biphasic decline, others showing a single-phase decline with a lower gradient, and some showing little to no change. For patients treated with ETV or LAM, the trend in HBV DNA reduction was consistent, showing a rapid initial decrease followed by a plateau, indicative of a biphasic decline (**Fig 2C**). Overall, while a decrease in HBcrAg was frequently observed, a reduction in HBsAg was not observed in most patients.

We employed our multiscale mathematical model, and used the derived linearized model equations (Eqs 14–16 and Eqs 23–25 and 27), under the assumption of negligible *de novo* infections under treatment [25,26,32–34], to evaluate clinical datasets to address the amount of cccDNA. For simplicity, we assumed the proportion of HBsAg produced from iDNA among HBeAg-negative patients is fixed to be 0.6 (i.e., HBsAg is dominantly from iDNA: *x* = 0.6) as reported in [4,5] because of the lowest AIC scores (see our sensitivity analysis on *x* in **Table A** in **S1 Text**), while that among HBeAg-positive patients is 0. All viral markers of all patients were simultaneously fitted using a nonlinear mixed-effect modeling approach (as illustrated for all individuals in **Figs 2A, 2B, 2C**, **and B** and **Note A** in **S1 Text**), which uses samples to estimate population parameters while accounting for inter-individual variation (**Tables B** and **C** in **S1 Text**). Note that some patients showed “biphasic” decay of HBcrAg because of precore mutations and basic core promoter mutations [29–31].

The model predicted that the decay rate of cccDNA varies among patients (**Table C** in **S1 Text**), showing a median half-life of cccDNA of around 7.7 years among patients treated with NAs (ETV or LAM) (**Fig 2D** and **Table 1**). PEG IFN-α significantly decreased the cccDNA half-life in all patients regardless of combination with NAs (**Fig 2D** and **Table 1**): the median values in patients achieving VR and PVR were 123 days (ranging minimum of 20 days to a maximum of 628 days; 20–628 days) and 756 days (61–1023 days) in HBeAg-positive and HBeAg-negative patients, respectively. The median values for non-VR and non-PVR patient groups were 520 days (74–1295 days) and 643 days (55–1420 days) for HBeAg-positive and HBeAg-negative patients, respectively. There were significant differences in the half-life between patients achieving VR and non-VR patients (*p*<0.01 by Mann-Whitney U tests), but not significant between patients achieving PVR and non-PVR. The rapid decline of cccDNA which is evident in HBeAg-positive patients who achieve VR may indicate a robust immune response [38]. We also examine the difference in the half-life of cccDNA after PEG IFN-α treatment according to *CC* or *CT* genotype on the IL28B SNP but no significant differences were observed (**Fig 2E**). The estimated half-lives of cccDNA in different subgroups of patients are summarized in **Table 1**.

**Table 1:** Estimated half-life of cccDNA.

Antiviral treatment	Median (day)	Min-max* (day)
NAs (ETV or LAM)-treated patient	2796	391−10820
PEG IFN-α-treated patient for VR of HBeAg positive	123	20−628
PEG IFN-α-treated patient for non-VR of HBeAg positive	520	74−1295
PEG IFN-α-treated patient for PVR of HBeAg negative	756	61−1023
• monotherapy	793	61−1023
•combinations with NAs	667	94−931
PEG IFN-α-treated patient for non-PVR of HBeAg negative	643	55−1420
•monotherapy	692	138−1420
•combinations with NAs	576	55−770

* Ranging minimum day to a maximum day

### Predicting the amount of intrahepatic cccDNA in chronically HBV-infected patients

**Fig 3:**
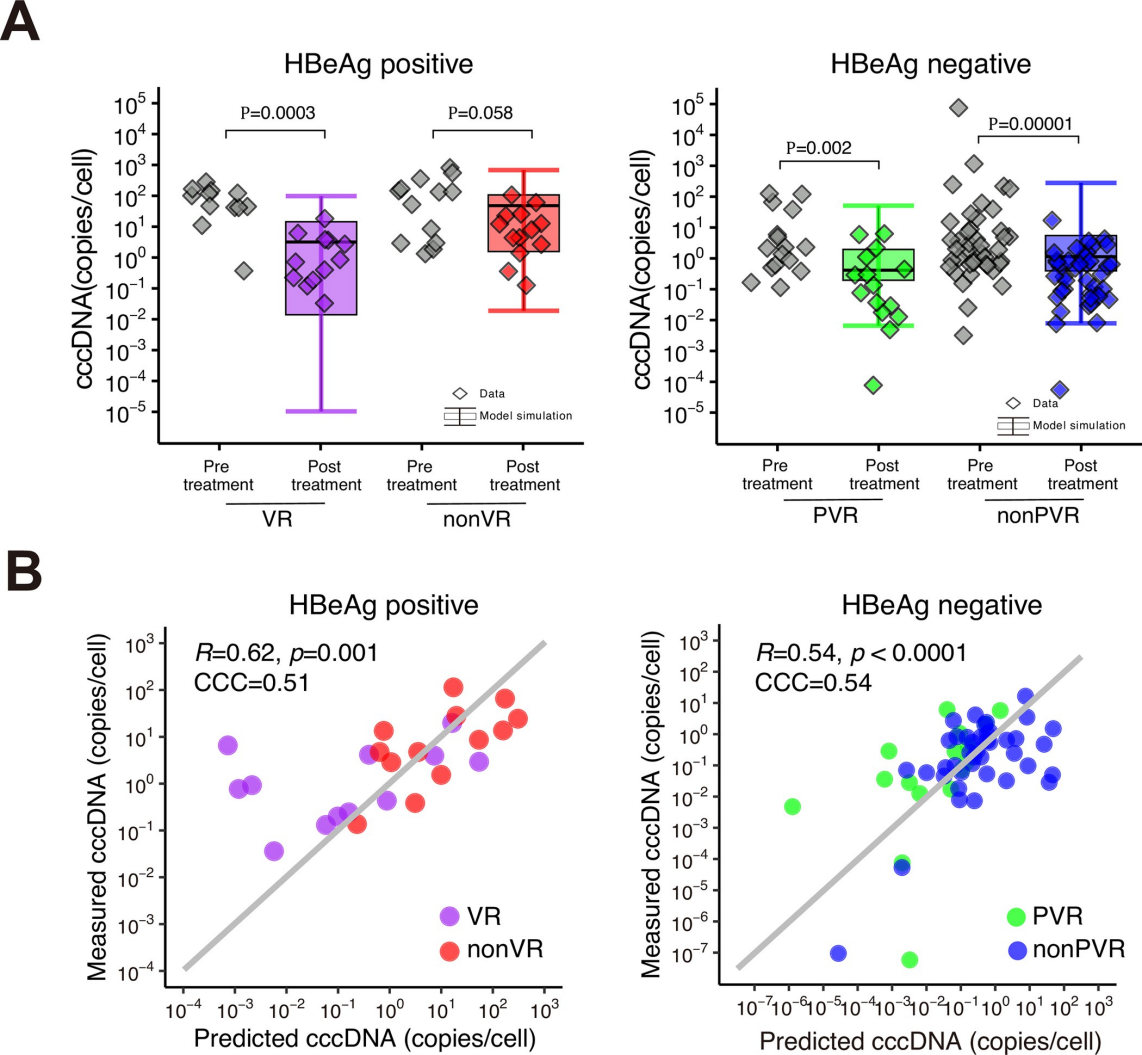
**Prediction of intrahepatic cccDNA in patients treated with PEG IFN-α: (A)** Group-level comparisons of simulated cccDNA per cell from Eq 27 with estimated parameters and the measured cccDNA before and at 48 weeks after treatment in hepatocytes of HBeAg-positive/negative and (P)VR/non-(P)VR patients treated with PEG IFN-α. Black line indicates the median; box and whiskers show the interquartile range (IQR) and 1.5xIQR, respectively. **(B)** Individual-level comparisons of predicted and measured cccDNA at post-treatment is shown. Correlations were calculated as Pearson correlation coefficients and Concordance Correlation Coefficient (CCC) was calculated as Lin’s concordance correlation coefficient for agreement between the two variables.

The quantity of cccDNA in patients prior to treatment was quantified with a median of 3.0 (95% CI 0.1–683.6) copies/cell, as determined by the first of paired liver biopsy samples, closely resembling previous findings (the gray marks in **Fig 3A**) [39–42]. Significant differences in the amount of cccDNA at the beginning of treatment were also observed between HBeAg-positive and HbeAg-negative patients (*p*<0.01 by Mann-Whitney U tests).

We here examined the validity of the estimates of the half-life of cccDNA under PEG IFN-α treatment calculated by Eq 27 by using the second of paired liver biopsy samples (pre-treatment and at 48 weeks [end of PEG IFN-α treatment]) and the estimated parameter summarized in **Table C in S1 Text**. Experimental measurement of cccDNA (using PSAD-treated liver samples) showed that the amounts of cccDNA were significantly reduced in the VR (HBeAg-positive) and PVR/non-PVR (HBeAg-negative) patients for PEG IFN-α, whereas the amounts in non-VR patients showed a minimal decrease (the colored marks in **Fig 3A**). In fact, the decay of the amount of cccDNA for all four groups (VR, non-VR, PVR, non-PVR) measured were within the range of simulated values by our mathematical model (the box and whiskers plots in **Fig 3A**), indicating that our mathematical model captured the cccDNA decay in both the HBeAg-positive and HBeAg-negative cohorts.

In addition to assessing the group difference, we also evaluated the correlation between individual-level measured and simulated cccDNA at 48 weeks after treatment in hepatocytes (**Fig 3B**). While our mathematical model well predicts the amount of cccDNA at the end of treatment, individual cccDNA amounts per se show a large variation regardless of group. In other chronic infectious diseases, genetic polymorphisms of patients are understood as one of the causes of such variation [43,44]. On the other hand, the role of immune activity and/or genetic polymorphisms in CHB patients might contribute to this, but this theory is still controversial and further study is necessary [45,46].

Note that in **Fig C** in **S1 Text**, we have also examined what the model predictions are like if not all three markers are used for parameter estimation. As shown, the cccDNA predictions using two markers for parameter estimation resulted in lower predictions compared to using all three markers. This supports our claim that using all three markers simultaneously leads to better predictions than using specific markers.

## Discussion

HBcrAg and HBV RNA have been proposed as surrogate markers for transcriptionally active cccDNA [15,17,18,20,47,48] and have been used to evaluate the antiviral effect of drugs to achieve functional cure. Recent clinical studies of new anti-HBV candidates such as HBV capsid inhibitors or small interfering RNAs (siRNAs) have measured HBV RNA as well as HBV DNA and viral antigens as surrogate markers [49],[50,51] reflecting cccDNA activity to study drug potential. However, these markers do not necessarily correlate with the amount of cccDNA because their values also reflect the transcriptional activity of cccDNA. A previous study estimated the turnover of cccDNA by monitoring the signature mutation (M204I/V) induced by lamivudine treatment in HBV RNA in the serum [52]. While this method is an innovative proposal, it is unclear whether the method will be useful in estimating the cccDNA amount and turnover in patients under PEG IFN-α therapy without the signature mutation. Developing a method to accurately estimate the turnover and quantity of cccDNA remains a significant challenge in the pursuit of achieving a complete cure, especially given the constraints of limited liver biopsy samples.

So far, mathematical models with several “compartmentalized stages” of intracellular HBV replication have been proposed [53,54]. Here, we employed our developed multiscale mathematical model explicitly including intracellular and extracellular HBV infection, described by age-structured PDEs, for quantifying HBV viral dynamics [21] and applied this model to the analysis of CHB patients. The amount of intrahepatic cccDNA and its dynamics are predicted by quantification of three serum viral markers—HBV DNA, HBsAg, and HBcrAg—in this multiscale model. The estimated half-life and reduction of intrahepatic cccDNA in PEG IFN-α treated patients were supported by clinical datasets including paired liver biopsy data for HBeAg-negative and HBeAg-positive cohorts. Our modeling approach allows the time-course estimation of the amount of cccDNA in CHB patients undergoing treatment without having to repeat liver biopsy. Note that in our previous study [21], we were unable to measure cccDNA in the liver without sacrificing the humanized mice. Therefore, we utilized the "average" cccDNA values from other mice as the baseline level for simulating our multiscale model. In contrast, with the availability of paired liver biopsies, we can now utilize the measured baseline cccDNA level for simulation and validate using the level at the end of treatment from the same patients. This significantly enhances the accuracy of our mathematical predictions.

Being able to estimate the turnover of intrahepatic cccDNA is important for evaluating and designing treatment for cccDNA clearance. The liver biopsy data indicated that PEG IFN-α reduced the amount of cccDNA but that it was difficult to eliminate cccDNA within 48 weeks of treatment consistent with previous reports that PEG IFN-α may target and reduce cccDNA, but the clinical effects of cccDNA clearance are seen in only a minority of CHB patients [55,56]. Given the clear reduction in cccDNA amount observed in the liver biopsy, especially in VR and PVR patients, and the accelerating cccDNA decay shown by our model, anti-viral activity of PEG IFN-α or treatment duration may not be sufficient for cccDNA elimination. From the perspective of side effects, clinical guidelines on the management of HBV infection in the EU, USA, and Japan specify a duration of PEG IFN-α treatment of 48 weeks. However, previous trials of extended-duration PEG IFN-α treatment in HBeAg-negative patients with poor IFN response [57] achieved significantly better VR and HBsAg loss [58–61], although extended PEG IFN-α treatment did not necessarily improve viral elimination in all patients. Our developed mathematical model enables us to quantify intrahepatic cccDNA dynamics in patients undergoing treatment, utilizing baseline cccDNA levels obtained from a single liver biopsy. This facilitates the identification of patients exhibiting a high cccDNA decay rate. For these patients, extending the duration of PEG IFN-α treatment might be effective. In the future, if evidence accumulates that extending the treatment duration increases the rate of achieving elimination of intrahepatic cccDNA, the benefit of extending treatment may outweigh the adverse effects.

Our mathematical model has a few assumptions underlying the intra- and inter-cellular HBV propagation. Firstly, we assumed negligible *de novo* infections under ETV and PEG IFN-α treatment because NAs and PEG IFN-α inhibit HBV replication by around 100% (i.e., *ε*≈1). The assumption may overestimate the mean half-life of cccDNA. After additional datasets on the time-course of the viral markers with different intensities of NAs and PEG IFN-α treatments become available, the inhibition rate can be determined more precisely and our estimation will be improved. For example, relaxing this assumption (i.e., *ε*<1) allows us to include hepatocyte proliferation in the target cell population as suggested by others [62]. The extended model may capture “non-standard” patterns of viral decline like a triphasic viral decline (e.g., IDJ19 in **Fig B** in **S1 Text**) and a flat-partial response (e.g., ID45 in **Fig B** in **S1 Text**). Second, we assumed that the cccDNA degradation rate under PEG IFN-α treatment, *d*_*IFN*_, includes different immune responsiveness that may develop during the treatment and also affect kinetics of clearance or alter cccDNA activity without clearance. However, the clearance mechanisms accompanying PEG IFN-α treatment in our mathematical model may be too simplified for the “all-in-one” cccDNA degradation, since there have been cases in which seroconversion of viral markers has been observed after completion of PEG IFN-α treatment [63,64]. This is presumed to be induced as a result of cccDNA degradation based on PEG IFN-α, but it is thought to be achieved by a more complex pathway involving immune cells rather than direct cccDNA degradation by PEG IFN-α, which is still an unclear mechanism. A quantitative (and time-dependent) mechanism of PEG IFN-α that alters intracellular HBV replication is necessary to improve our mathematical modeling in which variations due to the different immune responsiveness are taken into account. In addition, in a previous report [65], stochastic simulations using an agent-based model were used to analyze viral dynamics considering changes in the distribution of cccDNA abundance due to stochastic variation. Our model did not include these stochastic variations. The issue of noise that a deterministic model cannot capture is important, and reinterpreting our data with an agent-based model incorporating this noise could be a future study. Third, our model did not account for variability among hepatocytes, which is known to be spatially localized [66], and for hepatocyte variation or extra-hepatic sources of infection [67]. If these were accounted for and the relevant parameters could be estimated, it would enhance the utility of HBV infection dynamics investigations and provide a more accurate measure of cccDNA dynamics. However, the parameters added to the mathematical model might be difficult to estimate based on our limited data. Although the current simple but quantitative mathematical model successfully predicts the amount of cccDNA in patients from our extracellular viral markers, more precise mathematical modeling that improves these limitations will be beneficial for further development of CHB treatments.

We expect that the analysis of patient data that we did not access in this study will be very useful as a further verification of our mathematical model by combination of surrogate markers we proposed. Differences in the mechanism of action of HBV therapeutic drugs may affect the performance of surrogate markers. This study analyzed patient data in which PEG IFN-α, NAs, or both were administered. PEG IFN-α, due to its mechanism of action, reduces not only all markers of HBV DNA, HBsAg, and HBcrAg, but also cccDNA [35,68,69]. On the other hand, it must be taken into consideration that patients treated with anti-HBV drugs targeting specific viral markers may changes in the correlation between surrogate viral markers reflecting cccDNA activity. For example, capsid assembly modulators (CAMs) which is still in clinical trials, have been reported to weaken the correlation between viral markers in serum and cccDNA activity in patients treated with CAMs [70,71]. This finding indicates the possibility the need for improvement, as patients treated with anti-HBV drugs with different mechanisms of action than PEG IFN-α may not be able to track the dynamics of cccDNA, as demonstrated in our mathematical model.

In addition, analyzing patient data other than genotypes B and C, which we analyzed, is also important for verifying viral markers. Currently, genotype determination is not routinely performed before starting treatment, but genotype differences are likely to be an important factor in capturing the dynamics of cccDNA since they affect the sensitivity of the detected viral antigen and also the effects of anti-HBV drugs such as PEG IFN-α [72,73]. Previous reports have been shown that both host and viral factors influence IFN-α response during HBV infection. This population study is limited to Japanese and Thai patients with HBV infection. Therefore, in the Asian population, host genetic polymorphisms, including HLA and IL28B, are similar, and the patients are mainly infected with HBV genotype C, suggesting that this modeling prediction is suitable for the consideration of reactivity attributable to HBV factor. In contrast, HBV genotypes A and D occur frequently in Europe and North America, and genotype A is highly sensitive to IFN-α [74]. Furthermore, HLA class 1 and 2 genes associated with the pathogenesis, clearance of HBV, and clinical treatment outcome vary among ethnic populations [75]. In the future, the analysis of patients who received treatment with anti-HBV drugs not handled in this study and patients with different genotypes will be necessary to verify our mathematical model and improve the performance.

In summary, our multiscale mathematical model combined with an individual patient’s extracellular viral markers—HBsAg, HBcrAg, and HBV DNA—predicts the amount of intrahepatic cccDNA and opens new avenues to design a therapeutic strategy for achieving a complete HBV cure.

## Methods

### Ethics statement

Written informed consent was obtained from each patient and the study protocol conformed to the ethical guidelines of the Declaration of Helsinki and was approved by the appropriate institutional ethics review committees of each institute. The approval numbers for Nagoya City University Hospital, Teine-Keijinkai Hospital, and Nippon Medical School Chibahokusoh Hospital in Japan, and the King Chulalongkorn Memorial Hospital in Thailand are 60-00-0295, 2018–077, B-2020-263, and 70-00-0067, respectively.

### Study design and approval

The novel multiscale mathematical model describing intracellular viral propagation was applied to HBV-infected patients to predict the intracellular HBV dynamics. The CHB patient samples in this study were from 226 patients in total at Nagoya City University Hospital, Teine-Keijinkai Hospital, and Nippon Medical School Chibahokusoh Hospital in Japan and the King Chulalongkorn Memorial Hospital, Bangkok, in Thailand. The patients were classified into two clinical groups: (i) 199 CHB patients receiving PEG IFN-α monotherapy or PEG IFN-α in combination with NAs, which included 46 HBeAg-positive patients and 94 HBeAg-negative patients treated with PEG IFN-α alone and 59 HBeAg-negative patients treated with PEG-IFN-α and ETV combination and (ii) 27 patients receiving NAs (control group). Patients coinfected with HCV and/or HIV were excluded. The patient studies were not performed blinded. The study size was determined by the number of samples that were obtained from the cohort study and was not based on any power calculations.

### PEG IFN-α and NAs-treated HBV patients

Data were obtained from a total of 226 patients with CHB classified into two clinical groups: (i) patients treated with PEG IFN-α monotherapy or PEG IFN-α combination therapy with NAs and (ii) patients receiving NAs only, which were defined as the control group in this study (**Figs 1**, **A** and **B** in **S1 Text**).

Of the 226 patients, 199 patients (i) were treated with PEG IFN-α (180 μg/week) alone or ETV (0.5 mg/day) for 48 weeks and followed up for a minimum of 24 weeks after therapy. Of these 199 patients, 46 patients with HBeAg-positive CHB were seropositive for HBsAg and HBeAg for at least 6 months before therapy and the other 153 patients with HBeAg-negative CHB were seropositive for HBsAg for at least 6 months, negative for HBeAg, and positive for anti-HBe antibody. The remaining 27 patients (ii) were treated with ETV (0.5 or 1mg/day) or LAM (100 mg/day) continuously. Of these 27 patients, 15 patients with HBeAg-positive CHB were seropositive for HBsAg and HBeAg at study entry and the other 12 patients with HBeAg-negative CHB were seropositive for HBsAg at study entry, negative for HBeAg, and positive for anti-HBe antibody. *VR* was defined as HBeAg clearance and HBV DNA level <2,000 IU/ml at 48 weeks after treatment in HBeAg-positive CHB. *PVR* was defined as HBeAg clearance and HBV DNA level <2,000 IU/ml at 96 weeks after treatment in HBeAg-negative CHB.

Qualitative HBsAg, HBeAg, and anti-HBe in sera were measured by commercially available enzyme-linked immunosorbent assay kits (Abbott Laboratories, Chicago, IL, USA). HBsAg titers were quantified by use of Elecsys HBsAg II Quant reagent kits (Roche Diagnostics, Indianapolis, IN, USA). HBV DNA levels were quantified by use of the Abbott RealTime HBV assay (Abbott Laboratories, Chicago, IL, USA). The lower limit of detection of serum HBV DNA is 10 IU/ml. HBcrAg was measured by chemiluminescent enzyme immunoassay using a commercial assay kit (Fujirebio Inc., Tokyo, Japan). Paired liver biopsies were performed before and at the end of PEG IFN-α treatment for intrahepatic cccDNA analysis (week 0 and 48). After treatment with PSAD to digest linear genomic DNA and relaxed circular HBV DNA, intrahepatic cccDNA was determined by real-time PCR as described previously [36]. The beta-globin gene was used as an internal control and normalized for human genomic DNA in terms of copies/cell. Quantification of beta-globin was performed by a commercially available human genomic DNA kit (The LightCycler Control Kit DNA, Roche Diagnostics, Basel, Switzerland) [76].

### Statistical analysis

Transformation to the reduced model, data fitting, and parameter estimations are described in **Notes A** and **B** in **S1 Text** in detail. All analyses of samples were conducted using custom scripts in R and were visualized using RStudio. For comparisons between groups, Mann-Whitney U tests and t test were used. All tests were declared significant for *p*<0.01.

## Supporting information

S1 Text Fig A. Summary of HBV infection datasets. Fig B. HBV-infected patients treated with PEG IFN-α or ETV/LAM. Fig C. Prediction of intrahepatic cccDNA in patients treated with PEG IFN-α. Table A. AIC score with different *x*. Table B. Estimated population parameters and initial values for HBV-infected patients treated with PEG IFN-α or ETV/LAM. Table C. Estimated individual parameters and initial values for HBV-infected patients treated with PEG IFN-α or ETV/LAM. Note A. Transformation to a system of ODEs from a PDE multiscale model. Note B. Data fitting and parameter estimation.
(DOCX)
