# Supplementary material for: Prediction of cccDNA dynamics in hepatitis B patients by a combination of serum surrogate markers
Source: PLoS Comput Biol. 2025 Jan 9;21(1):e1012615. doi: 10.1371/journal.pcbi.1012615 (PMC11753647; doi:10.1371/journal.pcbi.1012615)
Supplement: S1 Text — Fig B. HBV-infected patients treated with PEG IFN-α or ETV/LAM. Fig C. Prediction of intrahepatic cccDNA in patients treated with PEG IFN-α. Table A. AIC score with different x. Table B. Estimated population parameters and initial values for HBV-infected patients treated with PEG IFN-α or ETV/LAM. Table C. Estimated individual parameters and initial values for HBV-infected patients treated with PEG IFN-α or ETV/LAM. Note A. Transformation to a system of ODEs from a PDE multiscale model. Note B. Data fitting and parameter estimation. (DOCX) [file pcbi.1012615.s001.docx]

**Supplementary Information**

Prediction of elimination of intrahepatic cccDNA in hepatitis B virus-infected patients by a combination of classic and emerging surrogate markers

Kwang Su Kim^1,2,†^, Masashi Iwamoto^1,3,†^,Kosaku Kitagawa^1,‡^, Hyeongki Park^1,‡^, Sanae Hayashi^4,‡^, Senko Tsukuda^5^, Takeshi Matsui^6^, Masanori Atsukawa^7^, Natthaya Chuaypen^8^, Pisit Tangkijvanich^8^, Lena Allweiss^9,10^, Takara Nishiyama^1^, Naotoshi Nakamura^1^, Yasuhisa Fujita^1^, Eiryo Kawakami^11,12^, Shinji Nakaoka^13^, Masamichi Muramatsu^3^, Kazuyuki Aihara^14^, Takaji Wakita^3^, Alan S. Perelson^15^, Maura Dandri^9,10^, Koichi Watashi^3,16,17,#,*^, Shingo Iwami^1,14,18,19,20,21,22,#,*^ & Yasuhito Tanaka^4^

^1^interdisciplinary Biology Laboratory (iBLab), Division of Natural Science, Graduate School of Science, Nagoya University, Nagoya 464-8602, Japan. ^2^Department of Science System Simulation, Pukyong National University, Busan, South Korea. ^3^Department of Virology II, National Institute of Infectious Diseases, Tokyo 162-8640, Japan. ^4^Department of Gastroenterology and Hepatology, Faculty of Life Sciences, Kumamoto University, Kumamoto 860-8556, Japan. ^5^Nuffield Department of Medicine, University of Oxford, Oxford OX3 7BN, UK. ^6^Center for Gastroenterology, Teine Keijinkai Hospital, Sapporo, Japan. ^7^Department of Gastroenterology and Hepatology, Nippon Medical School, Tokyo, Japan. ^8^Center of Excellence in Hepatitis and Liver cancer, Department of Biochemistry, Faculty of Medicine, Chulalongkorn University, Bangkok, Thailand. ^9^Department of Internal Medicine, University Medical Center Hamburg-Eppendorf, Hamburg, Germany. ^10^German Center for Infection Research (DZIF), Hamburg-Lübeck-Borstel-Riems partner sites, Germany. ^11^Artificial Intelligence Medicine, Graduate School of Medicine, Chiba University, Chiba 260-8670, Japan. ^12^Medical Sciences Innovation Hub Program, RIKEN, Yokohama, Kanagawa 230-0045, Japan. ^13^Faculty of Advanced Life Science, Hokkaido University, Sapporo 060-0810, Japan. ^14^International Research Center for Neurointelligence, The University of Tokyo Institutes for Advanced Study, The University of Tokyo, Tokyo, Japan. ^15^Theoretical Biology and Biophysics Group, Los Alamos National Laboratory, Los Alamos, NM 87545, USA. ^16^Research Center for Drug and Vaccine Development, National Institute of Infectious Diseases, Tokyo 162-8640, Japan. ^17^Department of Applied Biological Sciences, Faculty of Science and Technology, Tokyo University of Sciences, Chiba 278-8510, Japan. ^18^Institute of Mathematics for Industry, Kyushu University, Fukuoka, Japan 819-0395. ^19^Institute for the Advanced Study of Human Biology (ASHBi), Kyoto University, Kyoto 606-8501, Japan. ^20^NEXT-Ganken Program, Japanese Foundation for Cancer Research (JFCR), Tokyo 135-8550, Japan. ^21^Interdisciplinary Theoretical and Mathematical Sciences (iTHEMS), RIKEN, Wako 351-0198, Japan. ^22^Science Groove Inc., Fukuoka 810-0041, Japan.

**Fig A. Summary of** **HBV infection datasets:** Detailed data-sampling schedule for HBV-infected clinical patients.

**Fig B. HBV-infected patients treated with PEG IFN-α or ETV/LAM:** Decay characteristics are shown for extracellular HBV DNA, HBsAg, and HBcrAg in peripheral blood of HBeAg-positive patients treated with PEG IFN-α **(A)** with VR or **(B)** without VR (non-VR), HBeAg-negative patients treated with PEG IFN-α **(C)** with PVR or **(D)** without PVR (non-PVR), **(E)** HBeAg-negative patients treated with PEG IFN-α and ETV with PVR, **(F)** HBeAg-negative patients treated with PEG IFN-α and ETV without PVR (non-PVR), and **(G)** patients treated with ETV or LAM. Note that triangle point represents the detection limit (0 log10). Note that triangle points are the measured value as the detection limit (0 log10).


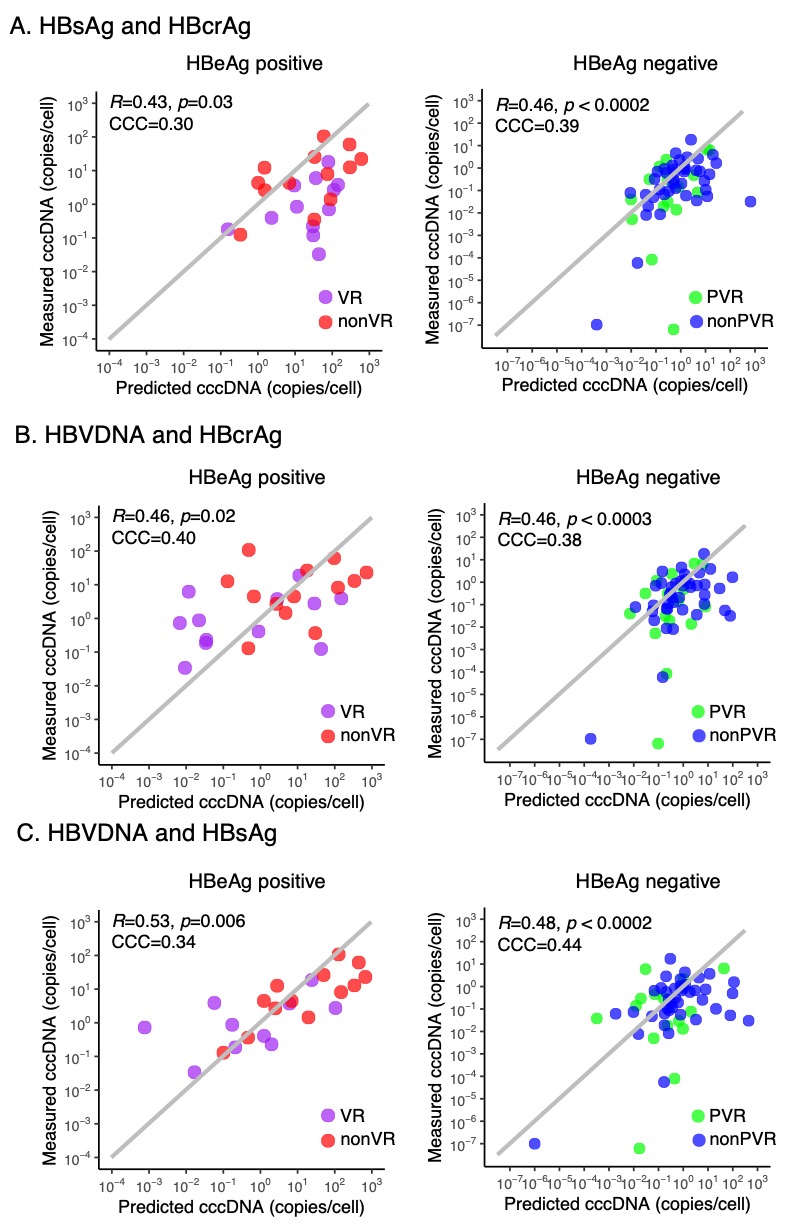


**Fig C. Prediction of intrahepatic cccDNA in patients treated with PEG IFN-α:** Individual-level comparisons of predicted and measured cccDNA at post-treatment are shown. Correlations were calculated as Pearson correlation coefficients and Concordance Correlation Coefficient (CCC) was calculated as Lin's concordance correlation coefficient for agreement between the two variables. Prediction of intrahepatic cccDNA with parameters estimated using **(A)** HBsAg and HBcrAg, **(B)** HBVDNA and HBcrAg, and **(C)** HBVDNA and HBsAg are shown, respectively.

**Table A. AIC score with different** $\boldsymbol{x}$

| **Proportion of HBsAg produced from iDNA among HBeAg-negative patients:** $\boldsymbol{x}$ | **AIC** |
| --- | --- |
| $0.3$ | $8166$ |
| $0.4$ | $8216$ |
| $0.5$ | $8021$ |
| $0.6$ | $8005$ |
| $0.7$ | $8257$ |
| $0.8$ | $8077$ |
| $0.9$ | $8108$ |

**Table B. Estimated population parameters and initial values for HBV-infected patients treated with PEG IFN-α or ETV/LAM**

| **Parameter or variable** | **Symbol** | **Unit** | **Value (S.E.)** | **95%CI*** |
| --- | --- | --- | --- | --- |
| Combined parameter^†^ | $f\alpha$(log10) | - | $-4.9(0.16)$ | $[-8.43,-1.42]$ |
| Inhibition rate of intracellular HBV DNA production | $\varepsilon$ | - | $0.98 (0.006)$ | $[0.84,0.99]$ |
| Inhibition rate of HBeAg (HBcrAg) production | $m$ | - | $0.41 (0.16)$ | $[0.36,0.46]$ |
| Proportion of HBsAg produced from integrated DNA | $x$ | - | $0.6$ (fixed) | - |
| Decay rate of infected cell | $\delta$(log10) | day^-1^ | $-3.72(0.01)$ | $[-4.3,-3.2]$ |
| Decay rate of infected cell with PEG IFN-α for HBeAg positive patients | $\delta_{IFN}$(log10) | day^-1^ | $-2.95(0.15)$ | $[-3.23,-2.64]$ |
| Decay rate of infected cell with PEG IFN-α for HBeAg negative patients | $\delta_{IFN}$(log10) | day^-1^ | $-2.55(0.16)$ | $[-3.10,-2.02]$ |
| Consumption rate of HBV DNA for virion | $\rho$ | day^-1^ | $0.16 (0.02)$ | $[0.14,0.18]$ |
| Degradation rate of cccDNA | $d$(log10) | day^-1^ | $-3.62(0.001)$ | $[-4.40,-3.00]$ |
| Degradation rate of cccDNA with PEG IFN-α | $d_{\mathrm{IFN}}$(log10) | day^-1^ | $-2.92(0.16)$ | $[-3.10,-2.74]$ |
| Initial value for extracellular HBV DNA for PEG IFN-α-treated patients | $V\left( 0 \right)$(log10) | IU/ml | $5.95(0.11)$ | $[5.77,6.12]$ |
| Initial value for extracellular HBsAg for PEG IFN-α-treated patients | $S\left( 0 \right)$(log10) | IU/ml | $3.47(0.06)$ | $[3.38,3.55]$ |
| Initial value for extracellular HBcrAg for PEG IFN-α-treated patients | $R\left( 0 \right)$(log10) | IU/ml | $5.48(0.15)$ | $[5.25,5.70]$ |
| Initial value for extracellular HBV DNA for ETV or LAM-treated patients | $V\left( 0 \right)$(log10) | IU/ml | $5.57(0.22)$ | $[5.17,5.97]$ |
| Initial value for extracellular HBsAg for ETV or LAM-treated patients | $S\left( 0 \right)$(log10) | IU/ml | $3.01(0.25)$ | $[2.77,3.26]$ |
| Initial value for extracellular HBcrAg for ETV or LAM-treated patients | $R\left( 0 \right)$(log10) | IU/ml | $5.07(0.40)$ | $[4.51,5.63]$ |

* Confidence interval.

^†^ Production rate of HBV DNA from cccDNA $\times$ Fraction of HBV DNA recycling for cccDNA.

**Table C. Estimated individual parameters and initial values for HBV-infected patients treated with** **PEG IFN-α or ETV/LAM**

| **Para**  **meter**  **or**  **variable** | Combined  parameter^†^ | | Inhibition rate  of intracellular  HBV DNA  production | | Inhibition  rate  of HBeAg  (HBcrAg)  production | Decay rate  of  Infected cell | Decay rate  of  Infected cell  with  PEG IFN-α | Consumption  rate  of  HBV DNA  for virion | Decay  rate  of cccDNA | Decay rate  of cccDNA  with  PEG IFN-α | Initial value  for  extracellular  HBV DNA | Initial value  for  extracellular  HBsAg | Initial value  for  extracellular  HBcrAg | Proportion  of HBsAg  produced  from  integrated DNA | Genotype |
| --- | --- | --- | --- | --- | --- | --- | --- | --- | --- | --- | --- | --- | --- | --- | --- |
| **Symbol** | $f\alpha$(log10) | | $\varepsilon$ | | $m$ | $\delta$(log10) | $\delta_{IFN}$(log10) | $\rho$ | $d$(log10) | $d_{\mathrm{IFN}}$(log10) | $V\left( 0 \right)$(log10) | $S\left( 0 \right)$(log10) | $R\left( 0 \right)$(log10) | $x$ | --- |
| **Unit** | --- | | --- | | --- | day^-1^ | day^-1^ | day^-1^ | day^-1^ | day^-1^ | IU/ml | IU/ml | IU/ml | --- | --- |
|  | | **Patient ID** | | | | | | | | | | | |  |  |
|  | | **PEG IFN-α-treated patient (HBeAg-positive VR)** | | | | | | | | | | | |  |  |
| 48 | -4.61341 | | 0.981343 | | 0.354893 | -3.4891723 | -2.71992 | 0.101733 | -2.8854104 | -2.17795 | 6.59162 | 2.66606 | 6.76639 | 0 | C |
| 19 | -5.11417 | | 0.981701 | | 0.414609 | -3.5822155 | -2.81305 | 0.0229969 | -3.3707115 | -2.66326 | 6.64194 | 3.65186 | 7.04706 | 0 | C |
| 5 | -4.86745 | | 0.980249 | | 0.99817 | -3.8746641 | -3.08386 | 0.334201 | -2.9910558 | -2.28356 | 8.37962 | 3.65528 | 8.91681 | 0 | B |
| 24 | -5.83272 | | 0.982289 | | 0.560555 | -3.5845436 | -2.81547 | 0.0458677 | -2.6908815 | -1.98343 | 6.62827 | 4.09949 | 7.64667 | 0 | C |
| 36 | -5.4542 | | 0.981957 | | 0.42922 | -3.7610728 | -2.99204 | 0.212586 | -3.6644815 | -2.95703 | 5.17772 | 3.86927 | 6.11375 | 0 | C |
| 46 | -5.11931 | | 0.981506 | | 0.938446 | -3.5151002 | -2.74491 | 0.124757 | -2.9562558 | -2.24876 | 6.53683 | 3.05441 | 7.07934 | 0 | C |
| 47 | -5.5058 | | 0.980651 | | 0.945881 | -3.3356817 | -2.56658 | 0.0883172 | -3.186939 | -2.47939 | 6.63429 | 3.92773 | 7.47759 | 0 | C |
| 39 | -5.72411 | | 0.982176 | | 0.361488 | -3.6522172 | -2.88297 | 0.114805 | -2.262266 | -1.55483 | 7.3207 | 2.52067 | 7.80739 | 0 | C |
| 43 | -5.02493 | | 0.981635 | | 0.358321 | -3.6717332 | -2.90256 | 0.0528296 | -2.2320683 | -1.52463 | 7.91162 | 4.83138 | 9.08449 | 0 | C |
| 49 | -4.8372 | | 0.981498 | | 0.550581 | -2.5129244 | -1.74322 | 0.0463039 | -3.5207038 | -2.81325 | 8.14322 | 4.52575 | 8.65229 | 0 | C |
| 51 | -4.93931 | | 0.98092 | | 0.367626 | -3.6485725 | -2.8785 | 0.0678079 | -2.3057759 | -1.5983 | 7.52492 | 4.75095 | 9.18892 | 0 | C |
| 55 | -5.91403 | | 0.982315 | | 0.325342 | -2.4566841 | -1.68809 | 0.0682501 | -3.5437538 | -2.8363 | 8.09187 | 3.55414 | 9.62613 | 0 | C |
| 63 | -5.84084 | | 0.982282 | | 0.378547 | -3.6545238 | -2.88544 | 0.101397 | -2.174536 | -1.46708 | 7.83574 | 3.5253 | 8.68849 | 0 | C |
| 64 | -6.08226 | | 0.98245 | | 0.9995 | -3.6280804 | -2.85892 | 0.0510762 | -2.4947949 | -1.78735 | 7.88251 | 3.91175 | 9.39099 | 0 | C |
| 71 | -5.57375 | | 0.982048 | | 0.490283 | -3.475142 | -2.70615 | 0.185677 | -3.3911593 | -2.68371 | 5.35356 | 3.75151 | 5.37793 | 0 | C |
|  | | **PEG IFN-α-treated patient (HBeAg-positive non-VR)** | | | | | | | | | | | |  |  |
| 60 | -5.20803 | | 0.981581 | | 0.993038 | -4.0003817 | -3.2288 | 0.0632413 | -3.6981246 | -2.99058 | 7.31534 | 4.15342 | 8.90822 | 0 | C |
| 65 | -4.58001 | | 0.981406 | | 0.973337 | -4.0742919 | -3.30494 | 0.217618 | -3.9789771 | -3.27153 | 6.81567 | 3.79663 | 8.60566 | 0 | C |
| 16 | -3.60941 | | 0.980648 | | 0.39357 | -3.4576718 | -2.68797 | 0.0558267 | -3.3589204 | -2.65146 | 8.44554 | 4.14275 | 8.35309 | 0 | C |
| 26 | -4.80398 | | 0.981474 | | 0.429828 | -3.525948 | -2.75666 | 0.0393926 | -3.3872838 | -2.67983 | 7.21383 | 3.54647 | 7.81713 | 0 | C |
| 29 | -3.33383 | | 0.980467 | | 0.407573 | -3.7572111 | -2.98743 | 0.117215 | -3.6763604 | -2.9689 | 7.11107 | 3.79094 | 7.38451 | 0 | C |
| 37 | -3.57968 | | 0.980628 | | 0.366023 | -4.0106439 | -3.24096 | 0.238121 | -3.9214204 | -3.21396 | 6.62232 | 3.28791 | 6.70314 | 0 | C |
| 42 | -3.05356 | | 0.980287 | | 0.439154 | -3.5959529 | -2.82609 | 0.0346436 | -3.4728126 | -2.76535 | 6.9384 | 3.64925 | 7.0985 | 0 | C |
| 58 | -2.70981 | | 0.980069 | | 0.423426 | -3.9137758 | -3.14378 | 0.165861 | -3.8322926 | -3.12483 | 7.72474 | 3.72082 | 7.85518 | 0 | C |
| 59 | -4.08263 | | 0.980966 | | 0.561326 | -3.5034155 | -2.73389 | 0.342009 | -3.443696 | -2.73624 | 7.50111 | 4.25122 | 7.94253 | 0 | C |
| 66 | -5.66953 | | 0.980227 | | 0.996045 | -2.9005229 | -2.13077 | 0.126497 | -3.5518788 | -2.84458 | 6.65092 | 3.0129 | 8.30776 | 0 | C |
| 81 | -2.20994 | | 0.979761 | | 0.396611 | -3.8340707 | -3.06386 | 0.143717 | -3.759697 | -3.05223 | 8.98471 | 4.66784 | 9.92578 | 0 | C |
| 13 | -3.05582 | | 0.980288 | | 0.373048 | -3.9750929 | -3.20523 | 0.16016 | -3.8892626 | -3.1818 | 8.46502 | 4.61199 | 9.38045 | 0 | C |
| 33 | -3.17801 | | 0.980366 | | 0.402054 | -3.6437124 | -2.87387 | 0.119796 | -3.5712604 | -2.8638 | 6.60139 | 4.1068 | 8.18235 | 0 | C |
| 45 | -2.97454 | | 0.980236 | | 0.407233 | -3.7486366 | -2.97872 | 0.178163 | -3.6734726 | -2.96601 | 8.14299 | 4.48085 | 8.71033 | 0 | B |
| 68 | -2.70148 | | 0.980064 | | 0.381348 | -3.7568688 | -2.98685 | 0.164951 | -3.6832826 | -2.97582 | 8.39951 | 4.51607 | 8.73218 | 0 | B |
| 32 | -5.35586 | | 0.981882 | | 0.392998 | -3.4804749 | -2.71134 | 0.0322977 | -3.1807515 | -2.4733 | 4.71896 | 2.59997 | 6.16504 | 0 | C |
| 34 | -6.22847 | | 0.98257 | | 0.342765 | -3.9629848 | -3.19423 | 0.0265093 | -3.8466871 | -3.13924 | 6.94598 | 3.639 | 7.27122 | 0 | C |
| 38 | -3.37177 | | 0.980494 | | 0.417465 | -2.9849181 | -2.21482 | 0.175642 | -3.4298382 | -2.72238 | 8.09699 | 3.61708 | 8.83193 | 0 | C |
| 40 | -4.61443 | | 0.981338 | | 0.398307 | -3.7087261 | -2.93941 | 0.0289343 | -3.5824438 | -2.87499 | 5.59502 | 3.59597 | 6.03084 | 0 | C |
| 53 | -3.0416 | | 0.980279 | | 0.375114 | -3.4239921 | -2.65405 | 0.128461 | -3.3925026 | -2.68504 | 5.7863 | 1.67648 | 8.70067 | 0 | C |
| 54 | -3.0456 | | 0.976829 | | 0.994885 | -3.7246361 | -2.94835 | 0.239365 | -3.4606251 | -2.75289 | 8.38284 | 4.09051 | 9.5552 | 0 | C |
| 62 | -3.06507 | | 0.980294 | | 0.359774 | -4.0009577 | -3.2311 | 0.170857 | -3.9144626 | -3.207 | 5.93486 | 3.47968 | 6.77935 | 0 | C |
| 67 | -4.1051 | | 0.980982 | | 0.399065 | -3.7666825 | -2.99718 | 0.173576 | -3.680816 | -2.97336 | 5.1993 | 3.65606 | 5.73745 | 0 | C |
| 69 | -4.8822 | | 0.98153 | | 0.409786 | -3.6547621 | -2.88552 | 0.0233227 | -3.4942638 | -2.78681 | 7.30237 | 4.34312 | 8.51011 | 0 | C |
| 70 | -4.12598 | | 0.980996 | | 0.408522 | -3.6297974 | -2.8603 | 0.0566333 | -3.5334482 | -2.82599 | 7.68552 | 3.96089 | 9.18212 | 0 | C |
| 35 | -2.60909 | | 0.980008 | | 0.453134 | -3.142234 | -2.37188 | 0.119018 | -3.3708704 | -2.66341 | 8.02425 | 4.8008 | 8.65301 | 0 | C |
| 41 | -4.88191 | | 0.98153 | | 0.385214 | -3.8433368 | -3.07411 | 0.0843547 | -3.7465238 | -3.03907 | 8.3261 | 4.65439 | 8.79818 | 0 | C |
| 44 | -2.63043 | | 0.980021 | | 0.388581 | -3.8864888 | -3.11647 | 0.14242 | -3.8062148 | -3.09875 | 7.80512 | 4.47102 | 8.7687 | 0 | C |
| 50 | -3.88167 | | 0.980773 | | 0.383881 | -3.5555931 | -2.78618 | 0.077641 | -2.7342236 | -2.02673 | 6.5183 | 2.66684 | 7.00315 | 0 | B |
| 52 | -3.17667 | | 0.980366 | | 0.408141 | -3.7666847 | -2.99685 | 0.140219 | -3.6876604 | -2.9802 | 7.82011 | 4.34336 | 8.33146 | 0 | C |
| 56 | -2.80274 | | 0.980128 | | 0.41999 | -3.4346765 | -2.66465 | 0.141542 | -3.4058326 | -2.69837 | 8.04317 | 4.57855 | 8.34409 | 0 | C |
|  | | **PEG IFN-α-treated patient (HBeAg-negative PVR)** | | | | | | | | | | | |  |  |
| 5 | -4.93301 | | 0.981567 | | 0.45999 | -2.6152787 | -1.84596 | 0.0977194 | -3.5619238 | -2.85447 | 6.46742 | 2.77797 | 4.46085 | 0.6 | C |
| 9 | -4.86544 | | 0.981518 | | 0.354242 | -2.535167 | -1.76581 | 0.074065 | -3.7955238 | -3.08807 | 5.20365 | 1.8457 | 5.62882 | 0.6 | C |
| 11 | -2.66906 | | 0.980044 | | 0.345416 | -2.4263465 | -1.65557 | 0.135986 | -3.7689738 | -3.06152 | 8.34674 | 3.64402 | 7.07248 | 0.6 | C |
| 31 | -5.31209 | | 0.981849 | | 0.403592 | -3.2847 | -2.51568 | 0.0501367 | -3.6372015 | -2.92975 | 4.70846 | 3.85896 | 3.69928 | 0.6 | C |
| 43 | -3.5218 | | 0.98059 | | 0.360673 | -2.5242774 | -1.7541 | 0.150044 | -3.7941838 | -3.08673 | 5.1335 | 3.47069 | 5.08035 | 0.6 | C |
| 45 | -4.90657 | | 0.981548 | | 0.365818 | -2.2483224 | -1.47931 | 0.0641374 | -3.7544338 | -3.04698 | 5.1086 | 2.9159 | 3.30918 | 0.6 | C |
| 64 | -5.00164 | | 0.98162 | | 0.362557 | -2.5437878 | -1.77463 | 0.284237 | -3.8154438 | -3.10799 | 4.84951 | 2.48307 | 3.52778 | 0.6 | C |
| 65 | -5.59764 | | 0.982067 | | 0.460375 | -3.0764689 | -2.30763 | 0.284199 | -3.5389815 | -2.83153 | 4.74525 | 2.98923 | 3.37335 | 0.6 | C |
| 67 | -5.29467 | | 0.981836 | | 0.53002 | -3.3653179 | -2.59628 | 0.219558 | -3.6430515 | -2.9356 | 5.31161 | 3.1771 | 2.95827 | 0.6 | C |
| 68 | -4.264 | | 0.981092 | | 0.403438 | -2.5800013 | -1.81021 | 0.17952 | -3.7588238 | -3.05137 | 6.13253 | 1.68885 | 3.69736 | 0.6 | B |
| 76 | -5.83111 | | 0.982249 | | 0.33441 | -2.6017679 | -1.83324 | 0.348147 | -3.8711138 | -3.16366 | 5.71564 | 2.93002 | 4.45897 | 0.6 | C |
| 77 | -6.03171 | | 0.976924 | | 0.988243 | -2.969214 | -2.20037 | 0.268093 | -3.7990933 | -3.09152 | 6.35058 | 2.67536 | 7.15921 | 0.6 | C |
| 78 | -5.71859 | | 0.982161 | | 0.368995 | -3.1612918 | -2.39256 | 0.246324 | -3.7353715 | -3.02792 | 4.7329 | 2.75872 | 4.77979 | 0.6 | C |
| 79 | -4.93003 | | 0.981538 | | 0.416496 | -3.3895029 | -2.62022 | 0.0545388 | -2.7787927 | -2.07135 | 6.39553 | 2.77325 | 5.72535 | 0.6 | C |
| 83 | -5.47374 | | 0.981971 | | 0.334764 | -2.833606 | -2.06479 | 0.158154 | -3.8277138 | -3.12026 | 4.83761 | 2.38862 | 5.03345 | 0.6 | C |
| 102 | -4.79586 | | 0.981468 | | 0.65561 | -3.0529189 | -2.28359 | 0.21416 | -3.6491738 | -2.94172 | 5.06198 | 3.23164 | 3.25371 | 0.6 | B |
| 105 | -5.25935 | | 0.981809 | | 0.364981 | -2.5556388 | -1.78668 | 0.272164 | -3.7629438 | -3.05549 | 5.14078 | 3.11404 | 5.58713 | 0.6 | C |
| 108 | -5.35413 | | 0.981881 | | 0.362205 | -3.0432184 | -2.27428 | 0.337109 | -3.7222715 | -3.01482 | 5.11613 | 3.20767 | 4.76238 | 0.6 | C |
| 109 | -4.41263 | | 0.981195 | | 0.365717 | -2.5886926 | -1.81907 | 0.295148 | -3.8583338 | -3.15088 | 5.11713 | 2.24667 | 4.4132 | 0.6 | C |
| 114 | -4.40366 | | 0.981189 | | 0.39759 | -2.634992 | -1.8654 | 0.234782 | -3.6679538 | -2.9605 | 5.36914 | 2.78524 | 3.9476 | 0.6 | C |
| 121 | -4.31339 | | 0.981126 | | 0.40494 | -2.5610616 | -1.79137 | 0.273718 | -3.8053838 | -3.09793 | 5.04575 | 2.39799 | 3.57942 | 0.6 | B |
| J20 | -6.30206 | | 0.982095 | | 0.515576 | -3.5803573 | -2.81314 | 0.175908 | -2.6489093 | -1.94144 | 8.80837 | 3.31117 | 7.69562 | 0 | --- |
| J25 | -5.11422 | | 0.981701 | | 0.467853 | -3.8992153 | -3.13006 | 0.17717 | -3.8025615 | -3.09511 | 6.20037 | 3.63962 | 5.63455 | 0 | --- |
| J38 | -4.32517 | | 0.981134 | | 0.424756 | -3.6312834 | -2.86186 | 0.0713403 | -3.539606 | -2.83215 | 4.4335 | 3.71133 | 5.38517 | 0 | --- |
| J40 | -4.21347 | | 0.981622 | | 0.987658 | -3.6888528 | -2.92145 | 0.154655 | -3.789046 | -3.08161 | 4.83462 | 4.11007 | 5.40074 | 0.6 | --- |
| J77 | -2.74572 | | 0.980092 | | 0.355624 | -2.9489589 | -2.17829 | 0.231208 | -3.876646 | -3.16919 | 4.1058 | 1.33326 | 3.51258 | 0.6 | --- |
| J79 | -4.9628 | | 0.981451 | | 0.338966 | -2.7846208 | -2.01607 | 0.30161 | -3.8439903 | -3.13649 | 3.40771 | 0.701187 | 3.68237 | 0.6 | --- |
| J80 | -5.68132 | | 0.982132 | | 0.364374 | -2.9126189 | -2.14391 | 0.249345 | -3.7861815 | -3.07873 | 4.99619 | 1.79567 | 3.59434 | 0.6 | --- |
|  | |  | | **PEG IFN-α and NAs treated patient (HBeAg-negative PVR)** | | | | | | | | | | | |
| 1 | -5.15166 | | 0.981729 | | 0.395612 | -3.0333715 | -2.26428 | 0.223666 | -3.6417938 | -2.93434 | 5.08542 | 2.33125 | 2.7397 | 0.6 | B |
| 6 | -5.70526 | | 0.98215 | | 0.417713 | -2.4598422 | -1.69109 | 0.100617 | -3.6992438 | -2.99179 | 6.69144 | 3.34447 | 5.61298 | 0.6 | C |
| 7 | -4.56511 | | 0.981303 | | 0.383812 | -2.7173719 | -1.9479 | 0.261733 | -3.5827838 | -2.87533 | 4.91785 | 2.50026 | 5.1157 | 0.6 | C |
| 25 | -5.06179 | | 0.981662 | | 0.436543 | -3.01578 | -2.24664 | 0.274639 | -3.6500938 | -2.94264 | 4.91684 | 3.32843 | 3.01925 | 0.6 | C |
| 54 | -5.49808 | | 0.98199 | | 0.415686 | -2.9334917 | -2.16464 | 0.303936 | -3.6898415 | -2.98239 | 4.7611 | 2.73501 | 3.2212 | 0.6 | C |
| 56 | -5.11105 | | 0.981698 | | 0.369711 | -2.6081647 | -1.83904 | 0.286545 | -3.8070438 | -3.09959 | 4.84054 | 1.72249 | 3.4128 | 0.6 | B |
| 60 | -5.50482 | | 0.981995 | | 0.398787 | -2.7195534 | -1.95074 | 0.273381 | -3.7725438 | -3.06509 | 5.10141 | 2.71485 | 3.72866 | 0.6 | C |
| 72 | -5.8954 | | 0.9823 | | 0.412447 | -3.5075799 | -2.73882 | 0.304629 | -3.7044693 | -2.99702 | 5.11627 | 3.37748 | 2.60017 | 0.6 | C |
| 75 | -5.64367 | | 0.982102 | | 0.365803 | -2.8136991 | -2.04498 | 0.212106 | -3.8117238 | -3.10427 | 5.09947 | 2.78523 | 3.5752 | 0.6 | C |
| 95 | -6.61271 | | 0.982917 | | 0.507944 | -3.3604955 | -2.59145 | 0.123099 | -2.8403483 | -2.13291 | 6.67132 | 3.96662 | 6.66948 | 0.6 | C |
| 96 | -6.15391 | | 0.982508 | | 0.447116 | -3.1099045 | -2.34142 | 0.329701 | -3.6612515 | -2.9538 | 5.32671 | 3.18378 | 2.68408 | 0.6 | C |
| 104 | -6.03097 | | 0.982409 | | 0.420644 | -3.1706961 | -2.40213 | 0.270647 | -3.6916615 | -2.98421 | 5.1003 | 3.30741 | 3.6824 | 0.6 | C |
| 110 | -4.96352 | | 0.98159 | | 0.367841 | -2.5182904 | -1.74913 | 0.301727 | -3.8360238 | -3.12857 | 4.84357 | 3.51296 | 3.91573 | 0.6 | C |
| 119 | -5.80261 | | 0.982227 | | 0.395813 | -3.4543931 | -2.68547 | 0.101228 | -3.3415393 | -2.63409 | 4.9384 | 3.36739 | 5.63285 | 0.6 | C |
|  | | **PEG IFN-α treated patient (HBeAg-negative non-PVR)** | | | | | | | | | | | |  |  |
| 2 | -4.64835 | | 0.981362 | | 0.400605 | -3.4630616 | -2.69372 | 0.215148 | -3.5934538 | -2.886 | 4.90921 | 3.76266 | 4.95307 | 0.6 | C |
| 3 | -4.56246 | | 0.981301 | | 0.375087 | -3.4787855 | -2.70938 | 0.0584049 | -3.7800838 | -3.07263 | 5.15088 | 2.82356 | 3.394 | 0.6 | B |
| 12 | -4.47269 | | 0.981237 | | 0.419219 | -3.4500761 | -2.68064 | 0.323931 | -3.6658538 | -2.9584 | 5.08851 | 3.77474 | 4.36795 | 0.6 | C |
| 14 | -4.21042 | | 0.981054 | | 0.417823 | -3.2547035 | -2.48504 | 0.051743 | -3.697816 | -2.99036 | 4.93128 | 2.2543 | 3.29778 | 0.6 | B |
| 15 | -4.67574 | | 0.981382 | | 0.38889 | -3.5003414 | -2.73101 | 0.343858 | -3.6547438 | -2.94729 | 5.89717 | 4.42977 | 3.89375 | 0.6 | C |
| 18 | -2.96169 | | 0.980228 | | 0.378443 | -2.6433995 | -1.87281 | 0.0912945 | -3.8083938 | -3.10094 | 6.1519 | 3.35808 | 4.81982 | 0.6 | B |
| 20 | -6.32794 | | 0.982651 | | 0.351633 | -3.3497432 | -2.5812 | 0.290804 | -3.5580293 | -2.85058 | 5.80687 | 4.51406 | 4.63786 | 0.6 | C |
| 22 | -4.62304 | | 0.981344 | | 0.377922 | -3.4442421 | -2.67488 | 0.0596581 | -3.7560838 | -3.04863 | 5.83172 | 3.56287 | 4.29577 | 0.6 | B |
| 24 | -3.51772 | | 0.980588 | | 0.366193 | -2.9730446 | -2.20288 | 0.154714 | -3.8323638 | -3.12491 | 5.29837 | 2.77127 | 4.60781 | 0.6 | C |
| 26 | -4.84075 | | 0.9815 | | 0.402975 | -3.0118653 | -2.24259 | 0.266587 | -3.6750338 | -2.96758 | 5.71157 | 3.91146 | 3.19081 | 0.6 | C |
| 29 | -3.08838 | | 0.980309 | | 0.383708 | -3.4098646 | -2.6397 | 0.164395 | -3.7105682 | -3.00311 | 5.94083 | 3.40244 | 5.43102 | 0.6 | C |
| 32 | -4.18536 | | 0.981037 | | 0.413281 | -3.3205717 | -2.55099 | 0.207183 | -3.552986 | -2.84553 | 5.57627 | 3.19873 | 3.6476 | 0.6 | C |
| 34 | -6.43519 | | 0.982847 | | 0.937567 | -2.8520581 | -2.08479 | 0.0859048 | -3.5851438 | -2.87769 | 7.41189 | 3.71668 | 5.84824 | 0.6 | C |
| 37 | -1.53822 | | 0.979361 | | 0.378308 | -3.6864612 | -2.9158 | 0.148775 | -3.8556348 | -3.14817 | 6.06042 | 3.51623 | 5.69774 | 0.6 | C |
| 38 | -5.92096 | | 0.982321 | | 0.38099 | -3.529915 | -2.76115 | 0.0852659 | -3.6789793 | -2.97153 | 4.76363 | 3.17139 | 4.57862 | 0.6 | C |
| 41 | -3.91092 | | 0.98085 | | 0.391583 | -3.4555553 | -2.68581 | 0.0760938 | -3.769836 | -3.06238 | 5.21057 | 2.8481 | 3.22018 | 0.6 | B |
| 48 | -4.17443 | | 0.98103 | | 0.409624 | -3.2912503 | -2.52162 | 0.174366 | -3.660436 | -2.95298 | 4.75128 | 3.08982 | 3.11349 | 0.6 | B |
| 49 | -4.00147 | | 0.980911 | | 0.365243 | -3.4733001 | -2.70368 | 0.0631245 | -3.612966 | -2.90551 | 5.90315 | 3.7291 | 4.49326 | 0.6 | B |
| 51 | -3.84021 | | 0.980802 | | 0.386388 | -3.3546108 | -2.58484 | 0.232416 | -3.639566 | -2.93211 | 5.62397 | 3.45047 | 5.45975 | 0.6 | C |
| 52 | -2.33198 | | 0.979836 | | 0.407423 | -3.5398741 | -2.76941 | 0.157452 | -3.7418026 | -3.03434 | 6.23803 | 3.78994 | 5.16627 | 0.6 | B |
| 53 | -2.56527 | | 0.979979 | | 0.355302 | -3.5241524 | -2.75376 | 0.944856 | -3.8022804 | -3.09482 | 7.3234 | 3.43969 | 6.9671 | 0.6 | C |
| 55 | -6.10854 | | 0.982472 | | 0.399623 | -3.7450428 | -2.97626 | 0.299381 | -3.7033971 | -2.99595 | 4.66163 | 2.94531 | 4.25689 | 0.6 | C |
| 58 | -5.35345 | | 0.98188 | | 0.433226 | -3.5293358 | -2.76028 | 0.229827 | -3.5310915 | -2.82364 | 5.215 | 3.27541 | 4.604 | 0.6 | C |
| 63 | -5.69188 | | 0.98214 | | 0.365553 | -3.3793283 | -2.61052 | 0.100485 | -3.6876515 | -2.9802 | 4.67757 | 3.80511 | 3.88746 | 0.6 | C |
| 71 | -5.48642 | | 0.981981 | | 0.383141 | -3.4753633 | -2.70643 | 0.295273 | -3.7432915 | -3.03584 | 5.18247 | 3.33514 | 3.31797 | 0.6 | B |
| 74 | -6.34884 | | 0.980892 | | 0.972477 | -3.6253292 | -2.85107 | 0.116128 | -3.7330738 | -3.02564 | 5.58914 | 4.42178 | 4.94841 | 0.6 | C |
| 84 | -5.56921 | | 0.982045 | | 0.364045 | -2.926863 | -2.15807 | 0.158456 | -3.7676815 | -3.06023 | 4.82178 | 3.07856 | 3.88572 | 0.6 | C |
| 86 | -1.49268 | | 0.979334 | | 0.367613 | -2.8033599 | -2.03175 | 0.139108 | -3.661366 | -2.95391 | 7.25515 | 4.24384 | 7.60489 | 0.6 | C |
| 88 | -7.06631 | | 0.98331 | | 0.580773 | -2.9738468 | -2.20551 | 0.125706 | -3.0071471 | -2.2997 | 7.34021 | 3.48475 | 4.5365 | 0.6 | C |
| 89 | -4.76803 | | 0.981448 | | 0.398729 | -3.1878159 | -2.41851 | 0.0379566 | -3.5597038 | -2.85225 | 5.197 | 3.21928 | 4.89466 | 0.6 | C |
| 90 | -5.92078 | | 0.98232 | | 0.448205 | -3.4657301 | -2.69696 | 0.218551 | -3.6147493 | -2.9073 | 4.7061 | 3.46059 | 2.87941 | 0.6 | C |
| 92 | -5.2253 | | 0.981783 | | 0.408979 | -3.5922487 | -2.82317 | 0.305717 | -3.7817915 | -3.07434 | 4.8861 | 3.18649 | 3.48335 | 0.6 | B |
| 98 | -3.16295 | | 0.980357 | | 0.434775 | -3.3388837 | -2.56876 | 0.132786 | -3.6209682 | -2.91351 | 5.287 | 3.31992 | 5.41483 | 0.6 | C |
| 100 | -4.80887 | | 0.981477 | | 0.474673 | -3.1504157 | -2.38112 | 0.341905 | -3.6717738 | -2.96432 | 6.12978 | 2.81358 | 4.18298 | 0.6 | B |
| 101 | -5.98938 | | 0.982376 | | 0.487427 | -3.4779336 | -2.70911 | 0.247844 | -3.4486693 | -2.74122 | 7.13172 | 3.51871 | 6.484 | 0.6 | B |
| 103 | -6.30487 | | 0.982633 | | 0.373705 | -3.6115098 | -2.84288 | 0.254432 | -3.6959671 | -2.98852 | 5.89995 | 4.30539 | 3.88465 | 0.6 | C |
| 112 | -2.8224 | | 0.98014 | | 0.38845 | -3.5750725 | -2.80489 | 0.0980998 | -3.7391304 | -3.03167 | 7.26885 | 3.66833 | 6.58138 | 0.6 | C |
| 117 | -5.13238 | | 0.981714 | | 0.390325 | -3.1458542 | -2.37675 | 0.276651 | -3.5443338 | -2.83688 | 4.82083 | 3.65133 | 4.39007 | 0.6 | C |
| 122 | -5.02278 | | 0.981633 | | 0.368659 | -3.6670857 | -2.89791 | 0.311108 | -3.7538938 | -3.04644 | 5.3389 | 3.50865 | 4.69751 | 0.6 | C |
| 124 | -5.60214 | | 0.98207 | | 0.387096 | -3.6098507 | -2.84092 | 0.223982 | -3.6839715 | -2.97652 | 4.75436 | 4.06683 | 3.55755 | 0.6 | C |
| 125 | -6.3341 | | 0.982657 | | 0.422963 | -3.6043447 | -2.83572 | 0.102213 | -3.6880171 | -2.98057 | 5.14488 | 3.80731 | 3.1031 | 0.6 | C |
| J18 | -5.52114 | | 0.982009 | | 0.876122 | -4.03679 | -3.26777 | 0.101018 | -3.9355593 | -3.22811 | 7.83781 | 4.40131 | 6.50335 | 0 | --- |
| J19 | -3.06431 | | 0.980226 | | 0.40856 | -2.789687 | -2.0191 | 0.133737 | -3.4895226 | -2.78206 | 8.49551 | 4.34444 | 8.13182 | 0 | --- |
| J21 | -4.75718 | | 0.98144 | | 0.38766 | -3.4021659 | -2.63286 | 0.250543 | -3.7425838 | -3.03513 | 5.55804 | 3.35092 | 3.83701 | 0.6 | --- |
| J22 | -4.78822 | | 0.981463 | | 0.38458 | -3.7133078 | -2.94403 | 0.330778 | -3.8314338 | -3.12398 | 5.45911 | 4.01266 | 3.20974 | 0.6 | --- |
| J23 | -4.85582 | | 0.981511 | | 0.397425 | -3.5322097 | -2.76296 | 0.247285 | -3.7310538 | -3.0236 | 5.62709 | 3.63866 | 4.51832 | 0.6 | --- |
| J26 | -2.85463 | | 0.98016 | | 0.420089 | -3.76577 | -2.99581 | 0.0827554 | -3.6853626 | -2.9779 | 6.98387 | 2.20957 | 7.01902 | 0 | --- |
| J27 | -4.9342 | | 0.981568 | | 0.46254 | -3.8053314 | -3.03612 | 0.0846545 | -3.7086438 | -3.00119 | 8.19939 | 3.94432 | 7.18321 | 0 | --- |
| J28 | -3.81971 | | 0.980788 | | 0.372758 | -3.0101593 | -2.24023 | 0.183487 | -3.7396038 | -3.03215 | 5.64556 | 3.80916 | 4.28312 | 0.6 | --- |
| J32 | -5.6242 | | 0.980454 | | 0.994028 | -3.3249803 | -2.55355 | 0.316056 | -3.3525421 | -2.64523 | 8.45191 | 3.09508 | 8.08317 | 0 | --- |
| J33 | -3.06204 | | 0.980292 | | 0.484249 | -3.9813752 | -3.21152 | 0.0674964 | -3.8920226 | -3.18456 | 9.14839 | 4.37278 | 8.38499 | 0 | --- |
| J34 | -4.70289 | | 0.981401 | | 0.478096 | -3.6288187 | -2.8595 | 0.370104 | -3.7627938 | -3.05534 | 5.80678 | 4.24705 | 3.90282 | 0.6 | --- |
| J35 | -3.43573 | | 0.980534 | | 0.405802 | -3.8695302 | -3.09979 | 0.0858135 | -3.7820404 | -3.07458 | 8.89995 | 4.19877 | 7.85469 | 0 | --- |
| J37 | -3.12773 | | 0.980334 | | 0.399116 | -3.9070875 | -3.13724 | 0.170928 | -3.8231826 | -3.11572 | 8.87814 | 4.3134 | 8.5205 | 0 | --- |
| J39 | -3.03106 | | 0.980272 | | 0.373093 | -4.0231429 | -3.25328 | 0.120065 | -3.9355226 | -3.22806 | 8.94714 | 4.64573 | 8.94149 | 0 | --- |
| J53 | -4.4209 | | 0.981201 | | 0.525088 | -3.0224387 | -2.25288 | 0.345115 | -3.7464938 | -3.03904 | 5.85459 | 2.71197 | 3.05615 | 0.6 | --- |
| J54 | -3.25354 | | 0.980415 | | 0.29598 | -3.9830178 | -3.21327 | 0.0279882 | -3.8806104 | -3.17315 | 9.27321 | 4.35487 | 8.27161 | 0 | --- |
| J55 | -5.21982 | | 0.98178 | | 0.991407 | -3.5577287 | -2.78865 | 0.249032 | -3.7245038 | -3.01705 | 6.49613 | 3.44577 | 6.32723 | 0.6 | --- |
| J56 | -2.13939 | | 0.979719 | | 0.363308 | -4.1055693 | -3.33553 | 0.157129 | -4.0190648 | -3.3116 | 6.1049 | 3.49833 | 5.83668 | 0 | --- |
| J57 | -3.99137 | | 0.980904 | | 0.425526 | -3.1732921 | -2.40358 | 0.223424 | -3.492826 | -2.78537 | 4.50627 | 3.36599 | 2.99762 | 0.6 | --- |
| J73 | -5.42351 | | 0.981933 | | 0.384932 | -3.7261196 | -2.95712 | 0.264587 | -3.8401215 | -3.13267 | 5.44938 | 3.35569 | 3.20695 | 0.6 | --- |
| J74 | -5.23917 | | 0.981794 | | 0.43555 | -3.4750944 | -2.70598 | 0.0468062 | -3.3337515 | -2.6263 | 7.8544 | 4.02655 | 6.7485 | 0 | --- |
| J75 | -1.96779 | | 0.979615 | | 0.372578 | -4.01147 | -3.24129 | 0.155493 | -3.929567 | -3.2221 | 8.95729 | 4.49221 | 7.2087 | 0 | --- |
| J76 | -5.00627 | | 0.981621 | | 0.378597 | -3.6731304 | -2.90397 | 0.0144933 | -3.4139415 | -2.70649 | 9.24071 | 4.51539 | 7.14795 | 0 | --- |
| J82 | -4.80903 | | 0.981477 | | 0.46479 | -3.4400904 | -2.67081 | 0.277025 | -3.7047238 | -2.99727 | 5.88315 | 3.09297 | 3.46568 | 0.6 | --- |
| J83 | -4.69718 | | 0.981397 | | 0.398714 | -3.6819587 | -2.91264 | 0.315084 | -3.7961138 | -3.08866 | 5.222 | 3.70825 | 3.27634 | 0.6 | --- |
|  | |  | | **PEG IFN-α and NAs treated patient (HBeAg-negative non-PVR)** | | | | | | | | | | | |
| 4 | -5.05956 | | 0.98166 | | 0.421078 | -3.6701406 | -2.90097 | 0.193049 | -3.6510138 | -2.94356 | 5.18986 | 2.71953 | 3.54005 | 0.6 | B |
| 8 | -4.6479 | | 0.981362 | | 0.37611 | -2.6849783 | -1.91556 | 0.309798 | -3.6275638 | -2.92011 | 5.06677 | 2.47585 | 4.77327 | 0.6 | C |
| 10 | -5.31698 | | 0.981852 | | 0.362467 | -3.0505367 | -2.28156 | 0.179914 | -3.5460115 | -2.83856 | 5.90813 | 3.78192 | 6.47195 | 0.6 | C |
| 13 | -5.41439 | | 0.981926 | | 0.398981 | -3.4056996 | -2.6367 | 0.292933 | -3.5326515 | -2.8252 | 4.77256 | 3.74792 | 4.229 | 0.6 | C |
| 16 | -5.4775 | | 0.981974 | | 0.399149 | -3.4497341 | -2.68076 | 0.252612 | -3.5759415 | -2.86849 | 4.76837 | 3.39765 | 4.81183 | 0.6 | C |
| 17 | -5.68324 | | 0.982145 | | 0.475437 | -2.7212565 | -1.95254 | 0.483455 | -3.4565193 | -2.74907 | 4.96442 | 2.88507 | 5.3957 | 0.6 | B |
| 19 | -5.13039 | | 0.981713 | | 0.377823 | -3.19733 | -2.42819 | 0.231929 | -3.1667115 | -2.45926 | 5.16532 | 3.37713 | 4.70551 | 0.6 | C |
| 21 | -5.11783 | | 0.981703 | | 0.447896 | -3.4299259 | -2.66074 | 0.0797124 | -3.0747415 | -2.36729 | 5.42688 | 3.78059 | 4.61344 | 0.6 | C |
| 27 | -5.57377 | | 0.982048 | | 0.373942 | -3.0964307 | -2.32763 | 0.0899518 | -3.7101315 | -3.00268 | 4.85927 | 3.2622 | 3.70841 | 0.6 | B |
| 28 | -4.49771 | | 0.981255 | | 0.375935 | -3.0074098 | -2.23792 | 0.225569 | -3.7061338 | -2.99868 | 5.26908 | 3.38236 | 5.25106 | 0.6 | C |
| 33 | -5.42971 | | 0.981938 | | 0.374356 | -3.1516135 | -2.38267 | 0.242559 | -3.5077315 | -2.80028 | 5.78968 | 3.52373 | 4.28381 | 0.6 | C |
| 35 | -6.32847 | | 0.982652 | | 0.4112 | -3.3923626 | -2.62372 | 0.313957 | -3.4423671 | -2.73492 | 6.64732 | 3.51761 | 6.18482 | 0.6 | C |
| 36 | -5.28199 | | 0.981826 | | 0.401688 | -3.118893 | -2.34985 | 0.218607 | -3.3834715 | -2.67602 | 5.22936 | 3.15863 | 4.59445 | 0.6 | C |
| 39 | -5.54786 | | 0.982028 | | 0.377742 | -3.0988811 | -2.33006 | 0.316806 | -3.7440115 | -3.03656 | 5.25549 | 3.89426 | 3.619 | 0.6 | C |
| 40 | -5.96987 | | 0.98236 | | 0.385644 | -3.3975159 | -2.62883 | 0.262614 | -3.6768693 | -2.96942 | 5.1942 | 4.26338 | 3.87612 | 0.6 | C |
| 42 | -5.86881 | | 0.982279 | | 0.405689 | -3.3664389 | -2.5976 | 0.317591 | -3.4012593 | -2.69381 | 4.86629 | 3.89113 | 4.54315 | 0.6 | C |
| 44 | -4.48449 | | 0.981246 | | 0.374195 | -2.7855983 | -2.01606 | 0.321122 | -3.7075738 | -3.00012 | 4.92536 | 2.08838 | 5.21807 | 0.6 | C |
| 46 | -5.10884 | | 0.981697 | | 0.366408 | -3.1329491 | -2.36385 | 0.290211 | -3.6911838 | -2.98373 | 5.2429 | 3.01386 | 3.96105 | 0.6 | B |
| 47 | -6.74809 | | 0.983009 | | 0.455328 | -3.3950326 | -2.62639 | 0.191319 | -3.2408649 | -2.53342 | 6.53545 | 3.6604 | 4.75167 | 0.6 | C |
| 50 | -6.11256 | | 0.982475 | | 0.415422 | -3.1832479 | -2.41472 | 0.223343 | -3.6761115 | -2.96866 | 5.18087 | 3.84508 | 3.3392 | 0.6 | C |
| 57 | -6.27509 | | 0.982608 | | 0.456911 | -3.4000308 | -2.63148 | 0.323464 | -3.6534293 | -2.94598 | 5.13839 | 3.65565 | 4.26851 | 0.6 | C |
| 59 | -6.2302 | | 0.982572 | | 0.444657 | -3.3354549 | -2.56682 | 0.229898 | -3.4814593 | -2.77401 | 5.24348 | 3.74526 | 5.5824 | 0.6 | C |
| 61 | -4.93702 | | 0.98157 | | 0.347798 | -3.4307963 | -2.66159 | 0.208168 | -3.6315138 | -2.92406 | 6.46673 | 3.12563 | 6.59157 | 0.6 | C |
| 62 | -4.99206 | | 0.981611 | | 0.42034 | -3.2595136 | -2.49032 | 0.241963 | -3.3253338 | -2.61788 | 5.35355 | 3.57493 | 4.67733 | 0.6 | C |
| 66 | -5.97046 | | 0.98236 | | 0.41494 | -3.1016763 | -2.3331 | 0.548762 | -3.6840315 | -2.97658 | 5.49322 | 3.85816 | 4.43062 | 0.6 | C |
| 69 | -5.12714 | | 0.98171 | | 0.408849 | -3.3773353 | -2.60818 | 0.267905 | -3.2123715 | -2.50492 | 4.82127 | 4.33269 | 4.77493 | 0.6 | C |
| 70 | -6.18585 | | 0.982534 | | 0.3913 | -3.3489698 | -2.58034 | 0.314786 | -3.5289593 | -2.82151 | 5.15324 | 3.67419 | 4.11883 | 0.6 | C |
| 73 | -6.27271 | | 0.982606 | | 0.381122 | -3.4812063 | -2.71263 | 0.105719 | -3.6771493 | -2.9697 | 5.22476 | 3.58406 | 5.41799 | 0.6 | C |
| 80 | -5.85403 | | 0.982267 | | 0.484793 | -3.2307442 | -2.46202 | 0.297375 | -3.5915615 | -2.88411 | 4.87127 | 3.04199 | 3.67865 | 0.6 | B |
| 81 | -4.8447 | | 0.981503 | | 0.575025 | -2.9895555 | -2.22027 | 0.29973 | -3.6880538 | -2.9806 | 5.70746 | 2.77082 | 3.90718 | 0.6 | B |
| 82 | -4.55497 | | 0.981296 | | 0.406209 | -3.2091134 | -2.43969 | 0.305662 | -3.6127038 | -2.90525 | 5.07694 | 3.04816 | 3.4011 | 0.6 | C |
| 85 | -5.52519 | | 0.982011 | | 0.378415 | -3.1391574 | -2.37027 | 0.092961 | -3.6096915 | -2.90224 | 4.77204 | 2.40973 | 5.82956 | 0.6 | C |
| 91 | -5.91672 | | 0.982317 | | 0.393218 | -3.467495 | -2.69873 | 0.291656 | -3.6267393 | -2.91929 | 4.6929 | 3.58456 | 2.56837 | 0.6 | C |
| 93 | -5.28752 | | 0.98183 | | 0.432611 | -3.3278681 | -2.55882 | 0.306908 | -3.5219315 | -2.81448 | 4.79152 | 4.02743 | 3.61478 | 0.6 | C |
| 94 | -5.65719 | | 0.982113 | | 0.380893 | -3.5260899 | -2.7572 | 0.316453 | -3.6583293 | -2.95088 | 4.90003 | 4.49682 | 4.37772 | 0.6 | C |
| 97 | -6.58043 | | 0.979358 | | 0.985789 | -3.5411134 | -2.76326 | 0.30705 | -3.6551245 | -2.94717 | 5.74051 | 3.62744 | 5.23212 | 0.6 | C |
| 106 | -4.43066 | | 0.981208 | | 0.380368 | -2.7525315 | -1.98296 | 0.306704 | -3.7535338 | -3.04608 | 4.93251 | 3.43652 | 3.14465 | 0.6 | C |
| 107 | -5.44852 | | 0.981952 | | 0.372941 | -3.5719239 | -2.80296 | 0.0365857 | -3.7260215 | -3.01857 | 4.30316 | 3.98061 | 3.23206 | 0.6 | C |
| 111 | -5.47275 | | 0.981971 | | 0.486263 | -3.3468935 | -2.57795 | 0.320245 | -3.6402215 | -2.93277 | 4.92971 | 3.24666 | 2.63563 | 0.6 | B |
| 113 | -6.80254 | | 0.983056 | | 0.370529 | -3.5600847 | -2.79159 | 0.265248 | -3.5516249 | -2.84418 | 6.32428 | 4.05496 | 4.48572 | 0.6 | C |
| 115 | -6.50095 | | 0.982797 | | 0.370033 | -3.5348379 | -2.76631 | 0.101478 | -3.6423671 | -2.93492 | 5.43924 | 3.19186 | 5.82035 | 0.6 | C |
| 116 | -5.86075 | | 0.982273 | | 0.477666 | -3.4540691 | -2.68522 | 0.185749 | -3.4810593 | -2.77361 | 4.73001 | 3.92328 | 3.73368 | 0.6 | C |
| 118 | -5.23623 | | 0.981792 | | 0.394982 | -3.5640762 | -2.795 | 0.194798 | -3.7240815 | -3.01663 | 4.82265 | 3.94283 | 4.15846 | 0.6 | C |
| 120 | -5.60805 | | 0.982075 | | 0.401892 | -3.3338244 | -2.56496 | 0.273109 | -3.6369115 | -2.92946 | 4.99747 | 3.10311 | 5.42577 | 0.6 | C |
| 123 | -5.43962 | | 0.981945 | | 0.510324 | -3.0745772 | -2.30545 | 0.299042 | -2.6075093 | -1.90006 | 7.21348 | 3.86642 | 6.90685 | 0.6 | C |
|  | | **NAs (ETV or LAM) set2** | | | | | | | | | | | |  |  |
| E01 | -5.12496 | | 0.981709 | | 0.563852 | -3.1605228 | - | 0.0382731 | -3.5881215 | - | 3.98546 | 1.16352 | 3.9464 | 0.6 | --- |
| E02 | -2.96873 | | 0.980244 | | 0.373872 | -2.5257798 | - | 0.0434147 | -3.6323238 | - | 6.45616 | 4.79511 | 8.66016 | 0.6 | --- |
| E03 | -4.68602 | | 0.981389 | | 0.471374 | -2.8716033 | - | 0.0772825 | -3.5526693 | - | 5.83228 | 3.41883 | 5.61115 | 0.6 | --- |
| E04 | -5.72593 | | 0.982167 | | 0.415227 | -3.3897581 | - | 0.367991 | -3.6056515 | - | 5.05599 | 2.96744 | 3.13843 | 0.6 | --- |
| E05 | -5.80244 | | 0.982227 | | 0.414464 | -3.1455289 | - | 0.0287194 | -3.5395571 | - | 4.95446 | 2.43289 | 4.76181 | 0.6 | --- |
| E06 | -5.03823 | | 0.979617 | | 0.992912 | -3.571391 | - | 0.0913864 | -3.7231071 | - | 5.65163 | 2.7319 | 7.48641 | 0 | --- |
| E07 | -4.7792 | | 0.981456 | | 0.510596 | -3.7819228 | - | 0.211146 | -3.6961482 | - | 5.55676 | 3.46932 | 3.3803 | 0 | --- |
| E08 | -5.56953 | | 0.983885 | | 0.968973 | -2.7599394 | - | 0.307797 | -3.6339523 | - | 5.33561 | 1.62433 | 4.03276 | 0.6 | --- |
| E09 | -4.43834 | | 0.981213 | | 0.420212 | -2.7957576 | - | 0.357195 | -3.6008515 | - | 5.64256 | 2.27773 | 3.56239 | 0.6 | --- |
| L01 | -4.67254 | | 0.981379 | | 0.393932 | -3.7233417 | - | 0.0931729 | -3.6312138 | - | 5.77636 | 3.15672 | 6.70144 | 0 | --- |
| L02 | -4.73003 | | 0.98142 | | 0.40191 | -3.7491793 | - | 0.0405443 | -3.6571338 | - | 5.29513 | 3.70955 | 5.64797 | 0 | --- |
| L03 | -5.10748 | | 0.981696 | | 0.406015 | -3.7033021 | - | 0.121541 | -3.6093315 | - | 5.60932 | 3.56212 | 5.0951 | 0 | --- |
| L04 | -4.95443 | | 0.981583 | | 0.404197 | -3.692026 | - | 0.130886 | -3.5966715 | - | 5.4617 | 3.7717 | 5.31944 | 0 | --- |
| L05 | -4.11096 | | 0.981083 | | 0.450586 | -2.5508008 | - | 0.0656693 | -3.5582338 | - | 6.07856 | 3.15221 | 5.46741 | 0.6 | --- |
| L06 | -4.32131 | | 0.981131 | | 0.449452 | -3.4787102 | - | 0.063999 | -3.7092982 | - | 5.04682 | 2.64512 | 4.77066 | 0.6 | --- |
| L07 | -4.85353 | | 0.978791 | | 0.994705 | -2.4628802 | - | 0.184098 | -4.1893672 | - | 6.92206 | 2.47131 | 6.24258 | 0.6 | --- |
| L08 | -5.7475 | | 0.982183 | | 0.431873 | -3.2571721 | - | 0.0856796 | -3.5703393 | - | 4.56752 | 3.26035 | 3.59123 | 0.6 | --- |
| L09-1 | -5.64074 | | 0.9821 | | 0.566079 | -2.8346805 | - | 0.0701759 | -3.5511493 | - | 4.76681 | 2.70921 | 3.61439 | 0.6 | --- |
| L09-2 | -5.70846 | | 0.982153 | | 0.402281 | -3.5994669 | - | 0.0641191 | -3.4856849 | - | 6.51195 | 3.14671 | 5.45433 | 0 | --- |
| L10-1 | -4.53034 | | 0.981278 | | 0.476588 | -3.3450659 | - | 0.460047 | -3.6369838 | - | 5.36629 | 2.42878 | 3.54041 | 0.6 | --- |
| L10-2 | -3.16757 | | 0.98036 | | 0.998232 | -1.9906947 | - | 0.27677 | -3.6452938 | - | 6.3126 | 2.09789 | 7.56489 | 0.6 | --- |
| L11 | -4.85462 | | 0.98151 | | 0.467818 | -3.7710491 | - | 0.0949429 | -3.683796 | - | 5.32408 | 3.94439 | 4.82693 | 0 | --- |
| L12 | -4.78208 | | 0.981458 | | 0.420001 | -3.57984 | - | 0.0593193 | -2.7510506 | - | 6.03003 | 3.48821 | 6.53272 | 0 | --- |
| L13 | -4.63006 | | 0.981349 | | 0.54273 | -3.5484346 | - | 0.0980745 | -3.4191705 | - | 5.87785 | 4.02739 | 7.50152 | 0 | --- |
| L14 | -5.41404 | | 0.983959 | | 0.980709 | -2.7182935 | - | 0.173539 | -3.168038 | - | 7.06749 | 3.30525 | 5.36857 | 0.6 | --- |
| L15 | -4.97998 | | 0.98162 | | 0.974429 | -3.407083 | - | 0.145495 | -3.3941011 | - | 5.94476 | 2.50584 | 4.30369 | 0.6 | --- |
| L16 | -4.87379 | | 0.981527 | | 0.422972 | -3.5778475 | - | 0.15472 | -3.7339282 | - | 6.54779 | 3.42915 | 3.28349 | 0.6 | --- |

^†^ Production rate of HBV DNA from cccDNA $\times$ Fraction of HBV DNA recycling for cccDNA.

**Note A. Transformation to a system of ODEs from a PDE multiscale model**

As we recently reported,[^1^](#_ENREF_1)^,^[^2^](#_ENREF_2) the multiscale PDE model, Eqs. (1-7), can be transformed into a mathematically identical set of ordinary differential equations as follows. Using the method of characteristics with initial and boundary conditions of $i\left( t,a \right)$, we transform Eq. (2) into

$$i\left( t,a \right)=\left\{ \begin{aligned} {e^{-\delta a}b\left( t-a \right)=e}^{-\delta a}\beta T\left( t-a \right)V\left( t-a \right), t>a, \\ e^{-\delta t}i_{0}\left( a-t \right), t<a. \end{aligned} \right. \left( S1 \right)$$

Then, $I\left( t \right)$ is evaluated as follows:

$$I\left( t \right)=\int_{0}^{t} e^{-\delta a}\beta T\left( t-a \right)V\left( t-a \right)da+\int_{t}^{\infty} e^{-\delta t}i_{0}\left( a-t \right)da=\int_{0}^{t} e^{-\delta\left( t-a \right)}\beta T\left( a \right)V\left( a \right)da+\int_{0}^{\infty} e^{-\delta t}i_{0}\left( a \right)da.$$

Since $\frac{d}{dt}\int_{0}^{t} f\left( t,a \right)da=f\left( t,t \right)+\int_{0}^{t} \frac{\partial f\left( t,a \right)}{\partial t}da$, differentiating $I\left( t \right)$ with respect to time $t$, we obtain the following ODE:

$$\frac{dI(t)}{dt}=\beta T\left( t \right)V\left( t \right)-\delta I\left( t \right).$$

Also, we consider the total amount of cccDNA $CC(t)$ and the total amount of rcDNA $DD\left( t \right)$, defined by

$$CC\left( t \right)=\int_{0}^{t} C\left( a \right)i\left( t,a \right)da, DD\left( t \right)=\int_{0}^{t} D\left( a \right)i\left( t,a \right)da,$$

respectively. Then we have

$$\frac{d}{dt}CC\left( t \right)=C\left( t \right)i\left( t,t \right)+\int_{0}^{t} C(a)\left( -\frac{\partial}{\partial a}i\left( t,a \right)-\delta i(t,a) \right)da=f\rho DD\left( t \right)-(d+\delta)CC(t),$$

$$\frac{d}{dt}DD\left( t \right)=D\left( t \right)i\left( t,t \right)+\int_{0}^{t} D(a)\left( -\frac{\partial}{\partial a}i\left( t,a \right)-\delta i(t,a) \right)da=\beta T\left( t \right)V\left( t \right)+\alpha CC\left( t \right)-\left( \rho+\delta\right)CC\left( t \right).$$

Therefore, the multiscale PDE model is described as the following equivalent system of ODEs:

$$\frac{dT\left( t \right)}{dt}=s-d_{T}T\left( t \right)-\beta T\left( t \right)V\left( t \right), \left( S2 \right)$$

$$\frac{dI\left( t \right)}{dt}=\beta T\left( t \right)V\left( t \right)-\delta I\left( t \right), \left( S3 \right)$$

$$\frac{dV\left( t \right)}{dt}=\left( 1-f \right)\rho DD\left( t \right)-\mu V\left( t \right), \left( S4 \right)$$

$$\frac{dS\left( t \right)}{dt}=\pi_{S}CC(t)+s_{i}I\left( t \right)-\sigma S\left( t \right), \left( S5 \right)$$

$$\frac{dR\left( t \right)}{dt}=\pi_{R}CC(t)-\sigma R\left( t \right), \left( S6 \right)$$

$$\frac{dCC\left( t \right)}{dt}=f\rho DD\left( t \right)-(d+\delta)CC(t), \left( S7 \right)$$

$$\frac{dDD(t)}{dt}=\alpha CC\left( t \right)-\left( \rho+\delta\right)CC\left( t \right)+\beta T\left( t \right)V\left( t \right). \left( S8 \right)$$

Note that Eqs. (*S*2-*S*8) will be further simplified for the purpose of data analysis depending on the antiviral treatment assumed (see main text).

**Note B. Data fitting and parameter estimation**

MONOLIX 2019R2 ([www.lixoft.com](http://www.lixoft.com)), a program for maximum likelihood estimation for a nonlinear mixed-effects model, was employed to fit the model, Eqs. (23-25), to extracellular HBV DNA, HBcrAg and HBsAg in patients PB receiving PEG IFN-α monotherapy or PEG IFN-α combination with ETV/LAM (**Fig B** in **S1 Text**). In addition, we fit the model, Eqs. (14-16), to extracellular HBV DNA, HBcrAg and HBsAg in patients PB receiving NAs (**Fig B** in **S1 Text**). We assumed that the clearance rates of extracellular HBV DNA and antigens were $\mu=0.5783$d^-1^ [^3^](#_ENREF_3) and $\sigma=0.1394$ d^-1^ [^4^](#_ENREF_4) as previously estimated, respectively. Nonlinear mixed-effects modelling approaches incorporate a fixed effect as well as a random effect describing the inter-patient variability in parameters. Including a random effect amounts to a partial pooling of the data between individuals to improve estimates of the parameters applicable across the population of cases. By using this approach, the differences between the above 3 different viral markers in PB in different individuals were not estimated explicitly, nor did we fully pool the data which would bias estimates towards highly sampled cases. In this method of estimation, each parameter estimate $\vartheta_{i} \left( =\vartheta{\times e}^{\pi_{i}} \right)$depends on the individual where $\vartheta$ is fixed effect, and $\pi_{i}$ is random effect with an assumed Gaussian distribution with mean 0 and standard deviation $\Omega$*.* Population parameters and individual parameters were estimated using the stochastic approximation expectation-approximation algorithm[^5^](#_ENREF_5) and empirical Bayes’ method[^6^](#_ENREF_6), respectively. Note that in the data fitting, as described in[^7^](#_ENREF_7), we simply set the numerical values of our mathematical model below the detection limit (1 log10) to the detection limit. We divided our datasets into five groups; [PEG IFN-α treated HBeAg positive patients achieving VR], [PEG IFN-α treated HBeAg positive patients showing non-VR], [PEG IFN-α treated HBeAg negative patients achieving PVR], [PEG IFN-α HBeAg negative treated patients showing non-PVR] and [ETV/LAM treated patients]. Estimated population parameters, initial values, and their interpatient variability are listed in **Fig F** in **S1 Text**.

**References**

1. Kitagawa, K.*, et al.* Mathematical Analysis of a Transformed ODE from a PDE Multiscale Model of Hepatitis C Virus Infection. *Bull Math Biol* **81**, 1427-1441 (2019).

2. Kitagawa, K., Nakaoka, S., Asai, Y., Watashi, K. & Iwami, S. A PDE multiscale model of hepatitis C virus infection can be transformed to a system of ODEs. *J Theor Biol* **448**, 80-85 (2018).

3. Whalley, S.A.*, et al.* Kinetics of acute hepatitis B virus infection in humans. *J Exp Med* **193**, 847-854 (2001).

4. Goyal, A., Liao, L.E. & Perelson, A.S. Within-host mathematical models of hepatitis B virus infection: Past, present, and future. *Curr Opin Syst Biol* **18**, 27-35 (2019).

5. Kuhn, E. & Lavielle, M. Maximum likelihood estimation in nonlinear mixed effects models. *Computational statistics & data analysis* **49**, 1020-1038 (2005).

6. Pinheiro, J. & Bates, D. *Mixed-effects models in S and S-PLUS*, (Springer Science & Business Media, 2006).

7. Lavielle, M., Samson, A., Karina Fermin, A. & Mentré, F. Maximum likelihood estimation of long-term HIV dynamic models and antiviral response. *Biometrics* **67**, 250-259 (2011).
